# Correlation Between Periprocedural Myocardial Infarction, Mortality, and Quality of Life in Coronary Revascularization Trials: A Meta-analysis

**DOI:** 10.1016/j.jscai.2023.100591

**Published:** 2023-04-03

**Authors:** Mario Gaudino, Antonino Di Franco, Arnaldo Dimagli, Giuseppe Biondi-Zoccai, Mohamed Rahouma, Roberto Perezgrovas Olaria, Giovanni Soletti, Gianmarco Cancelli, David Chadow, John A. Spertus, Deepak L. Bhatt, Stephen E. Fremes, Gregg W. Stone

**Affiliations:** a Department of Cardiothoracic Surgery, Weill Cornell Medicine, New York, New York; b Department of Medical-Surgical Sciences and Biotechnologies, Sapienza University of Rome, Latina, Italy; c Mediterranea-Cardiocentro, Napoli, Italy; d Department of Biomedical and Health Informatics, University of Missouri-Kansas City, Kansas City, Missouri; e Saint Luke’s Mid America Heart Institute, Kansas City, Missouri; f Brigham and Women’s Hospital Heart & Vascular Center, Harvard Medical School, Boston, Massachusetts; g Division of Cardiac Surgery, Schulich Heart Centre, Department of Surgery, Sunnybrook Health Sciences Centre, University of Toronto, Toronto, Ontario, Canada; h The Zena and Michael A. Wiener Cardiovascular Institute, Icahn School of Medicine at Mount Sinai, New York, New York

**Keywords:** coronary artery bypass surgery, percutaneous coronary intervention, periprocedural myocardial infarction

## Abstract

**Background:**

The prognostic importance of periprocedural myocardial infarction (pMI) and its inclusion in the composite outcomes of coronary revascularization trials are controversial. We assessed whether pMI is a surrogate for all-cause or cardiac mortality and quality of life (QoL) outcomes in coronary revascularization trials.

**Methods:**

All randomized trials comparing percutaneous coronary intervention vs coronary artery bypass grafting (MEDLINE, EMBASE, Cochrane Library) were identified. Trials were included if they reported data for pMI and mortality. Trial-level associations between pMI and all-cause or cardiac mortality and QoL were assessed using the coefficient of determination (*R*^*2*^). The criterion for surrogacy was set at 0.7. Subgroup analyses based on pMI definition and on key clinical/procedural variables were performed.

**Results:**

Twelve trials were included (11,549 patients; weighted mean follow-up: 5.6 years). There was a positive correlation between pMI and all-cause mortality (slope, 1.81; 95% CI, 1.00-2.63; *R*^*2*^ = 0.72). In the trials that defined pMI as a rise in cardiac biomarkers >5 times the upper reference limit, pMI positively correlated with both all-cause (slope, 2.07; 95% CI, 1.00-3.14; *R*^*2*^ = 0.93) and cardiac mortality (slope, 0.70; 95% CI, 0.20-1.19; *R*^*2*^ = 0.87); no such relationships were present in trials that used a lower biomarker threshold. An inverse correlation was found between pMI and long-term changes in the Short Form Health Survey Physical Component score (slope, -4.66; 95% CI, -5.75 to -3.57; *R*^*2*^ =0.99).

**Conclusions:**

In the published coronary revascularization trials, pMI defined by larger biomarker elevations was associated with subsequent mortality and reduced QoL. These findings suggest that large pMI should be included as an outcome measure in coronary revascularization trials.

## Highlights

• Periprocedural myocardial infarction defined by larger biomarker elevations is associated with subsequent mortality.
• Periprocedural myocardial infarction defined by larger biomarker elevations is associated with reduced quality of life.
• Large periprocedural myocardial infarction should be included as an outcome measure in coronary revascularization randomized controlled trials.

## Central Illustration

**Figure:**
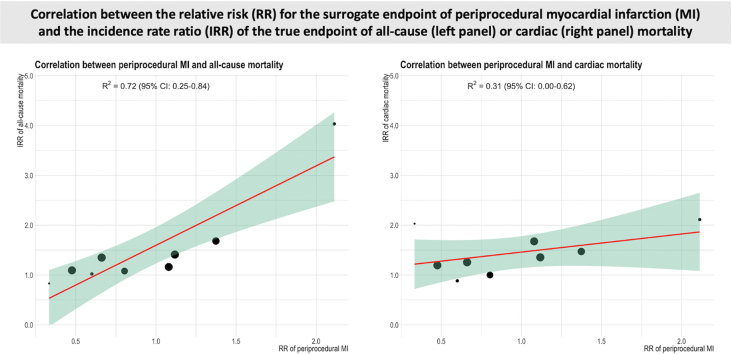

## Introduction

Myocardial infarction (MI) has traditionally been included in the primary composite outcome of randomized controlled trials (RCTs) comparing percutaneous coronary intervention (PCI) and coronary artery bypass surgery (CABG).^1^, ^2^, ^3^ MI is biologically related to coronary artery disease (CAD) and has historically been associated with an adverse prognosis in observational studies of patients with CAD,^4^ leading to general acceptance of its use as surrogate outcome of mortality.^5^

However, recently there has been controversy over the definition and prognostic importance of nonfatal MI, and in particular of periprocedural MI (pMI).^6^^,^^7^ Advances in cardiac imaging and laboratory medicine have enabled detection of progressively smaller degrees of myonecrosis,^8^ increasing the frequency of both periprocedural and nonprocedural MI.^9^ Whether smaller MIs (and particularly pMIs) affect survival and quality of life (QoL) is controversial. In addition, some degree of periprocedural myocardial injury is inherent with both PCI and CABG, and the association of pMI with mortality has been reported to be weaker than for nonprocedural MIs and varies with the pMI definitions used.^10^, ^11^, ^12^

To better evaluate the prognostic association of pMI in the modern era, we analyzed recent randomized trials comparing PCI with CABG to assess whether pMI correlates with subsequent all-cause or cardiac mortality and QoL. Moreover, we assessed the potential modifier effect of the pMI definition used in the different trials, and of other key clinical and procedural variables.

## Methods

### Ethics statement

Institutional review approval was waived, as this was a study-level meta-analysis of published reports and does not contain patient data.

### Search strategy

This study was performed in accordance with the Preferred Reporting Items for Systematic Reviews and Meta-analyses (PRISMA) guidelines.^13^ The analytic protocol was defined a priori but not registered.

A medical librarian performed comprehensive searches to identify all RCTs comparing PCI with CABG. Searches were performed in January 2022 using the following databases: Ovid MEDLINE (1946 to present), Ovid EMBASE (1974 to present), and the Cochrane Library (Wiley). The full search strategy is provided in the Appendix.

Trials were considered for inclusion if they compared PCI with either drug-eluting or bare-metal stents to CABG for the treatment of CAD and reported data for all-cause mortality and pMI. All articles were reviewed and analyzed for data by 2 independent authors (G.C. and R.P.O.) and disagreements were resolved by a third author (M.G.). The quality of the included trials was assessed using the Cochrane Collaboration’s tool.

Extracted variables included study years, number of participating centers, location, patient number, patient characteristics including age, sex, body mass index, cardiovascular risk factors (smoking status, diabetes, hypertension, dyslipidemia), peripheral vascular disease, carotid artery disease, left ventricular ejection fraction (LVEF), New York Heart Association class, European System for Cardiac Operative Risk Evaluation (EuroSCORE), SYNergy between percutaneous coronary intervention with TAXus and cardiac surgery (SYNTAX) score, extent of CAD, procedural characteristics (number of coronary lesions, completeness of coronary revascularization, number of stents, stent characteristics, bifurcation technique, use of intravascular ultrasound, type and number of surgical grafts, off-pump CABG technique, use of intraoperative graft assessment), details of medical therapy, mean/median follow-up time, and trial definition of pMI. Extracted outcomes included all-cause mortality, cardiac mortality (where available), and pMI. Details of the definitions of pMI used in each trial are provided in Supplemental Table S1.

Studies that included the measurement of patient QoL were also identified. The most consistently reported QoL measures were extracted at the longest follow-up available for each study: the Seattle Angina Questionnaire Angina Frequency scale and the physical and mental component scores of the 36-item and 12-item Short Form Health Survey (SF-36 and SF-12).

### Statistical analyses

To evaluate whether pMI is a surrogate for all-cause or cardiac mortality, we used the method adopted by Buyse et al^14^ In order to generate a graphic representation of the association between pMI and all-cause or cardiac mortality, the relative risk (RR) for pMI (the putative surrogate) was graphed on the x-axis with the incidence rate ratio (IRR) for all-cause or cardiac mortality (the primary outcome) graphed on the y-axis, with each trial serving as a unique data point. The RR for pMI was either extracted from the data published for each trial, if provided, or calculated from the number of events when not readily available. The RR represents the ratio of the rates of pMI in the PCI group over the rates of pMI in the CABG group. Similarly, the IRR was calculated by linearizing the incidence of mortality over the length of follow-up in each study. A horizontal line with slope = 0 indicates no association, a positive slope indicates some degree of positive association, and a negative slope indicates an inverse association between pMI and mortality. We attempted to determine the surrogate treatment effect, defined as the maximum value of the RR for pMI that needs to be observed in a trial to conclude a significant effect on all-cause or cardiac mortality. Because positive correlation does not necessarily meet the more stringent criteria for surrogacy, trial-level surrogacy of pMI for all-cause or cardiac mortality was assessed by generating a coefficient of determination, *R*^*2*^ (with 95% CIs), between the RRs for pMI and IRR for all-cause and cardiac mortality using a linear regression weighting each trial by the inverse of the variance of the RR pMI.^15^ The *R*^*2*^ values (corresponding to the explained variation) fall between 0 and 1.00, with 0 indicating the absence of surrogacy and 1.00 indicating perfect surrogacy. The CI for *R*^*2*^ was obtained using the R “confintr” package.^16^ The threshold for validating pMI as a surrogate for all-cause or cardiac mortality was set at 0.7. This threshold was determined a priori to limit post hoc biases. Prespecified subgroup analyses based on trial era (performed before or after year 2000), cardiac biomarker threshold used in MI definition (≤5 times vs >5 times the upper reference limit [URL]), follow-up duration, stent type, type of revascularization (PCI or CABG) and coronary anatomy (multivessel vs left main disease) were performed.

As sensitivity analysis, we assessed the impact of study-level factors such as age, sex, diabetes, and LVEF on the correlation between pMI and all-cause and cardiac mortality using the R package “mixmeta.”

To investigate the association between pMI and QoL, the changes in QoL from baseline to the longest available follow-up were calculated as mean difference (MD), and the correlation between the MD and the log-transformed IRR for pMI was calculated. To generate a graphic representation of the association between MD and pMI, the RRs for pMI were graphed on the x-axis and MDs were graphed on the y-axis. The coefficient of determination between the MD and the RR for pMI was then calculated using linear regression weighted by the inverse of the variance of the RR pMI.

All statistical analyses were performed in R, version 4.1.1 (R Foundation for Statistical Computing) within RStudio.

## Results

### Trials included and patient characteristics

Searches retrieved 7177 results. Following deduplication, 5433 citations were screened, for which 12 RCTs met the inclusion criteria and were included in the meta-analysis (Table 1).^17^, ^18^, ^19^, ^20^, ^21^, ^22^, ^23^, ^24^, ^25^, ^26^, ^27^, ^28^, ^29^, ^30^ The PRISMA flowchart outlining the study selection process is reported in Supplemental Figure S1.

**Table 1:** Details of the randomized trials included in the analysis.

Trial	No. of centers	Location	Study period	No. of patients randomized	Mean follow-up (years)	Type of pMI definition used (Details in Supplemental Table S1)
BARI Investigators,^17^ 2007	18	United States	1988-1991	1829 (PCI: 915, CABG: 914)	10.4	Protocol definition
Blazek et al,^18^ 2013	1	Germany	1997-2001	220 (PCI: 110, CABG: 110)	10.3	First Universal Definition of MI
Boudriot et al,^19^ 2011	3	Germany	2003-2009	201 (PCI: 100, CABG: 101)	1	Protocol definition
Kapur et al,^20^ 2010 (CARDia)	24	United Kingdom	2002-2007	510 (PCI: 256, CABG: 254)	1	Protocol definition
Stone et al,^21^ 2019 (EXCEL)	126	Europe, North America, Asia, South America	2010-2014	1905 (PCI: 948, CABG: 957)	5	Protocol definition
Fearon et al,^22^ 2022 (FAME 3)	48	Europe, United States	2014-2020	1500 (PCI:757, CABG: 743)	1	Protocol definition
Farkouh et al,^23^^,^^24^ 2012, 2019 (FREEDOM)	140	United States	2005-2010	1900 (PCI: 953, CABG: 947)	3.8, 7.5	Protocol definition
Hong et al,^25^ 2005	1	Korea	March 2003-Nov 2003	189 (PCI:119, CABG:70)	0.5	Not reported definition
Holm et al,^26^ 2020 (NOBLE)	36	Europe	2008-2015	1184 (PCI: 592, CABG: 592)	4.9	Protocol definition
Mohr et al,^27^ 2013; Thuijs et al,^28^ 2019 (SYNTAX)	85	Europe, United States	2005-2007	1800 (PCI: 903, CABG: 897)	5, 10	Protocol definition
Blazek et al,^29^ 2015	1	Germany	2003-2007	130 (PCI: 65, CABG: 65)	1	First Universal Definition of MI
Kamalesh et al,^30^ 2013 (VA CARDS)	22	United States	2006-2010	207 (PCI: 104, CABG: 103)	2	Protocol definition

CABG, coronary artery bypass grafting; PCI, percutaneous coronary intervention; MI, myocardial infarction; pMI, periprocedural myocardial infarction.

A total of 11,549 patients were included (PCI: 5813; CABG: 5736); RCTs were conducted from 1988 to 2020. The number of patients in the individual trials ranged from 130 to 1905. Weighted mean follow-up was 5.6 years (range 0.5-10.4 years). The mean age of the patients ranged from 61 to 67.5 years. The prevalence of women ranged from 1% to 35.9% and the prevalence of diabetes ranged from 15% to 100%.

Eight trials relied on creatine kinase-myocardial band (CK-MB) for pMI diagnosis and 1 trial on troponin. Five trials used a definition of pMI that included a rise in cardiac biomarkers >5 times the URL while 4 trials used a lower biomarker threshold (ie, ≤5 times the URL). Three trials did not provide details of the biomarker definition used.

Patient characteristics, procedural details, and details of medical therapy are summarized in Supplemental Tables S2-S4. The Cochrane Collaboration’s tool for assessing risk of bias for the assessment of the quality of the individual trials is shown in Supplemental Table S5.

### Correlation between pMI and mortality

There was a positive correlation between pMI and all-cause mortality (slope, 1.81; 95% CI, 1.00-2.63) with coefficient of determination above the prespecified threshold for surrogacy (*R*^*2*^ = 0.72) (Central Illustration and Table 2). No significant correlation was found between pMI and cardiac mortality (slope, 0.42; 95%CI, -0.14 to 0.98; *R*^*2*^ = 0.31) (Central Illustration and Table 2).

**Central Illustration:**
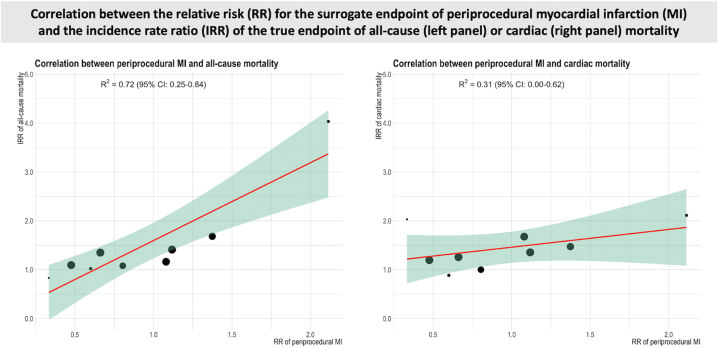
**Correlation between the relative risk for the surrogate end point of periprocedural myocardial infarction and the incidence rate ratio of the true end point of all-cause (left panel) or cardiac (right panel) mortality.** The green area represents the 95% CI for the regression line (red), and circle sizes are proportionate to the number of observations. IRR, incident rate ratio; MI, myocardial infarction; RR, relative risk.

**Table 2:** Correlations between periprocedural myocardial infarction and all-cause and cardiac mortality.

Analysis	Studies	Patients	Regression formula	Slope (95% CI)	*R*^*2*^ (95% CI)
pMI and all-cause mortality	12	11,549	-0.40 + 1.81 × RR, *P* = .001	1.81 (1.00-2.63)	0.72 (0.25-0.84)
pMI and cardiac mortality	9	10,657	1.07 + 0.42 × RR, *P* = .12	0.42 (-0.14 to 0.98)	0.31 (0.001-0.62)

pMI, periprocedural myocardial infarction; RR, relative risk.

In the trials that defined a pMI as a rise in cardiac biomarkers >5 times the URL, pMI positively correlated with both all-cause mortality (slope, 2.07; 95% CI, 1.00-3.14; *R*^*2*^ = 0.93) and cardiac mortality (slope, 0.70; 95% CI, 0.20-1.19; *R*^*2*^ = 0.87). No such relationships were present when pMI was defined using a lower biomarker threshold (Supplemental Table S6 and Supplemental Figures S2-S3). When the analysis was limited to the 8 trials that used CK-MB as cardiac biomarker, the correlation between pMI and cardiac mortality was confirmed (slope, 0.70; 95% CI, -0.11 to 1.51; *R*^*2*^ = 0.87) and the correlation with all-cause mortality was stronger (slope, 2.08; 95% CI, 0.56-3.61; *R*^*2*^ = 0.95) (Supplemental Table S6 and Supplemental Figures S4-S5). The results of the subgroup analyses were consistent with the results of the main analysis (Supplemental Table S6 and Supplemental Figures S6-S15). Sensitivity analyses confirmed the results of the main analysis (Supplemental Table S7).

### Correlation between pMI and QoL

**Figure 1:**
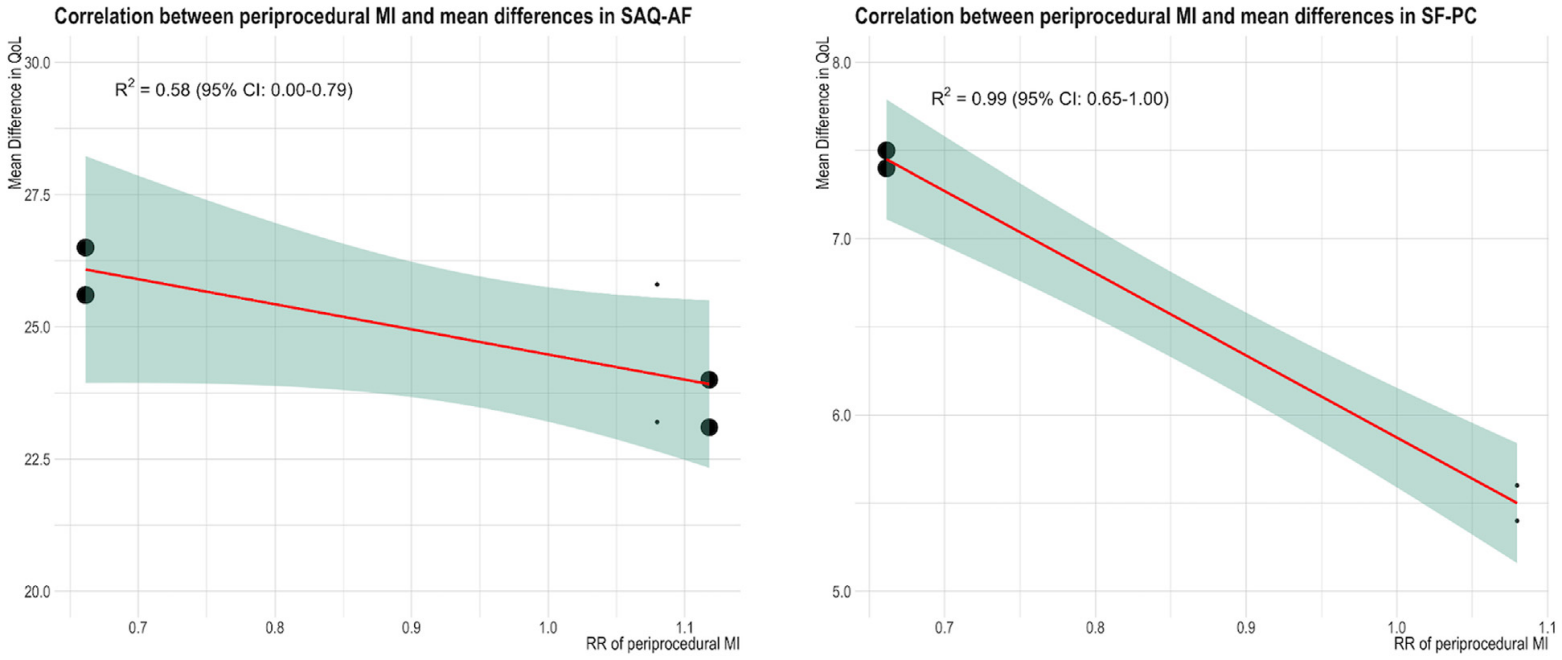
**Correlation between periprocedural myocardial infarction and changes in quality of life (QoL).** The green area represents the 95% CI for the regression line (red), and circle sizes are proportionate to the number of observations. Changes in QoL from baseline to the longest available follow-up are represented on the y-axis as mean differences. SAQ-AF, Seattle Angina Questionnaire Angina Frequency (left panel); SF-PC, Short Form Health Survey Physical Component score (right panel); MI, myocardial infarction; RR, relative risk.

Four trials reported QoL data. There was an inverse association between pMI and changes in the Short Form Health Survey Physical Component score (slope, -4.66; 95% CI, -5.75 to -3.57; *R*^*2*^ = 0.99) (Figure 1 and Table 3). No other correlations between pMI and QoL domain changes were found, including mental health and improvements in angina frequency.

**Table 3:** Correlations between periprocedural myocardial infarction and changes in quality of life.

Analysis	Studies	Patients	Regression formula	Slope (95% CI)	*R*^*2*^ (95% CI)
pMI and SAQ-AF	3	5605	29.06 - 4.59 × RR, *P* = .08	-4.52 (-9.86 to 0.81)	0.58 (0.002-0.79)
pMI and SF-PC	2	3705	10.53 - 4.66 × RR, *P* = .003	-4.66 (-5.75 to -3.57)	0.99 (0.65-1.00)

pMI, periprocedural myocardial infarction; RR, relative risk; SAQ-AF, Seattle Angina Questionnaire Angina Frequency; SF-PC Short Form Health Survey Physical Component score.

## Discussion

In the present analysis of 12 RCTs comparing PCI with CABG, we found that pMI was correlated with all-cause mortality. This relationship was present only with pMI defined by large biomarker elevations (>5 times URL), and such pMI events were also associated with cardiac mortality. There was also a correlation between pMI and the physical component of QoL assessments. These results were consistent in sensitivity analyses accounting for era of enrollment, follow-up duration, stent type, coronary anatomy, and type of procedure, as well as when adjusting for age, sex, diabetes, and LVEF at the trial level.

MI is biologically linked with CAD and has historically been associated with mortality in registries of patients with CAD.^4^ Based on this rationale, it has traditionally been assumed that nonfatal MI is a surrogate for mortality and that treatments that reduce these periprocedural events would also reduce the latter. In fact, pMI has generally been used in the composite outcome of contemporary randomized trials comparing PCI and CABG in order to increase efficiency and reduce sample size.^5^

Recently, there has been considerable controversy on the prognostic importance of nonfatal MI, and, in particular, of pMI. In individual studies the association with mortality has generally been weaker for pMI as compared with nonprocedural MI,^10^, ^11^, ^12^ and its frequency has been highly variable based on the MI definition used.^7^^,^^31^, ^32^, ^33^, ^34^ Moreover, as pMI is a relatively infrequent event, individual studies may have been underpowered to detect even moderate associations with mortality. Some authors have even suggested that pMI should not be included in the composite outcome of RCTs of coronary revascularization,^35^ and some recent trials have included only spontaneous MI in their primary outcome.^3^ A previous report on trials that tested interventions to treat or prevent CAD published during the last 50 years failed to show a correlation between nonfatal MI and all-cause or cardiovascular mortality.^5^ That analysis, however, included heterogenous interventions, so the generalizability of the results to PCI vs CABG trials was uncertain. In addition, subanalysis based on the timing of MI was not presented, and it is likely that survivorship bias for nonprocedural MI may have affected the overall results (as the time of follow-up after nonprocedural MIs may have been too short to show a correlation).

Of note, some prior studies in which the extent of procedural myonecrosis was not considered have reported a weak relationship between pMI and survival, likely driven by smaller pMIs. For example, in a recent metanalysis of 25 RCTs including a total of 19,806 patients with clinically stable CAD randomized to revascularization plus medical therapy vs medical therapy alone, by meta-regression the absolute difference in pMI was not significantly correlated with the absolute difference in cardiac mortality (β = -0.14; *P* = .16).^36^ In a pooled analysis of patient-level data from 9081 patients with chronic coronary syndromes undergoing PCI, Silvain et al^37^ found a significant association between post-PCI troponin elevation and 1-year mortality, but only with a >3-fold increase in troponin above the URL, with a continuous increase in mortality until a 25-fold elevation; major procedural myocardial injury defined as a post-PCI elevation in cardiac biomarkers ≥5 times URL was significantly associated with 1-year mortality (adjusted odds ratio, 2.29; 95% CI, 1.32-3.97; *P* = .004). Our data confirm and extend these results, suggesting that pMIs defined by larger biomarker elevations after PCI and CABG are associated with subsequent mortality and reduced QoL and should thus be included as an end point in revascularization trials.

Several limitations of our analysis warrant mention. The patient populations in the included RCTs were heterogenous, and there were differences in follow-up durations and outcome assessments. Although we have evaluated the effect of the biomarker threshold on the association of pMI with mortality, we could not provide more granular data on the effect of the evidence of ischemia (eg, ST-segment changes) in addition to the biomarker increase or of the individual pMI definitions used. Similarly, we were not able to assess the risk of pMI in those patients in whom postprocedural biomarkers were not collected. In addition, without patient-level data, it is likely that there is confounding with risk factors for both pMI and mortality for which we could not fully adjust. For example, trials in which the risk for pMI was high might reflect enrollment of higher-risk patients with greater comorbidities and complex coronary anatomy, rather than a causal effect of pMI. Only 1 trial defined pMI using troponin elevation; our results thus apply mostly to the use of CK-MB as a biomarker to assess periprocedural myonecrosis. Only 4 studies were present that reported QoL data; the relationship between pMI and reduced QoL are thus less robust than that between pMI and mortality. The present study was not designed to determine whether the same biomarker threshold to define pMI should be used after PCI and CABG. However, one study using individual patient data found similar relative hazards between the same multiples of CK-MB and troponin elevations after PCI and CABG.^7^ Finally, the surrogacy threshold of 0.7 that we used has not been formally validated, and different cut-offs have been used by others.^38^ In light of these reasons, the findings of the present study should be considered hypothesis-generating.

In conclusion, in this analysis of 12 PCI vs CABG RCTs, pMI was associated with mortality and reduced QoL, especially extensive myonecrosis as defined by a CK-MB elevation >5 times URL. The present study supports the inclusion of pMI defined by larger biomarker elevations as an outcome measure in coronary revascularization trials.

## Declaration of competing interest

Antonino Di Franco has consulted for Servier, Novo Nordisk, and is an Advisory Board Member for Scharper. Gianmarco Cancelli is supported by the Enrico and Enrica Sovena Foundation. Deepak L. Bhatt discloses the following relationships – Advisory Board: Bayer, Boehringer Ingelheim, Cardax, CellProthera, Cereno Scientific, Elsevier Practice Update Cardiology, Janssen, Level Ex, Medscape Cardiology, Merck, MyoKardia, NirvaMed, Novo Nordisk, PhaseBio, PLx Pharma, Regado Biosciences, Stasys; Board of Directors: AngioWave (stock options), Boston VA Research Institute, DRS.LINQ (stock options), Society of Cardiovascular Patient Care, TobeSoft; Chair: Inaugural Chair, American Heart Association Quality Oversight Committee; Data Monitoring Committees: Acesion Pharma, Assistance Publique-Hôpitaux de Paris, Baim Institute for Clinical Research (formerly Harvard Clinical Research Institute, for the PORTICO trial, funded by St. Jude Medical, now Abbott), Boston Scientific (Chair, PEITHO trial), Cleveland Clinic (including for the ExCEED trial, funded by Edwards), Contego Medical (Chair, PERFORMANCE 2), Duke Clinical Research Institute, Mayo Clinic, Mount Sinai School of Medicine (for the ENVISAGE trial, funded by Daiichi Sankyo; for the ABILITY-DM trial, funded by Concept Medical), Novartis, Population Health Research Institute; Rutgers University (for the NIH-funded MINT Trial); Honoraria: American College of Cardiology (Senior Associate Editor, Clinical Trials and News, ACC.org; Chair, ACC Accreditation Oversight Committee), Arnold and Porter law firm (work related to Sanofi/Bristol-Myers Squibb clopidogrel litigation), Baim Institute for Clinical Research (formerly Harvard Clinical Research Institute; RE-DUAL PCI clinical trial steering committee funded by Boehringer Ingelheim; AEGIS-II executive committee funded by CSL Behring), Belvoir Publications (Editor in Chief, Harvard Heart Letter), Canadian Medical and Surgical Knowledge Translation Research Group (clinical trial steering committees), Cowen and Company, Duke Clinical Research Institute (clinical trial steering committees, including for the PRONOUNCE trial, funded by Ferring Pharmaceuticals), HMP Global (Editor in Chief, *Journal of Invasive Cardiology*), *Journal of the American College of Cardiology* (Guest Editor; Associate Editor), K2P (Co-Chair, interdisciplinary curriculum), Level Ex, Medtelligence/ReachMD (CME steering committees), MJH Life Sciences, Oakstone CME, Piper Sandler, Population Health Research Institute (for the COMPASS operations committee, publications committee, steering committee, and USA national co-leader, funded by Bayer), Slack Publications (Chief Medical Editor, Cardiology Today’s Intervention), Society of Cardiovascular Patient Care (Secretary/Treasurer), WebMD (CME steering committees), Wiley (steering committee); Other: Clinical Cardiology (Deputy Editor), NCDR-ACTION Registry Steering Committee (Chair), VA CART Research and Publications Committee (Chair); Research Funding: Abbott, Acesion Pharma, Afimmune, Aker Biomarine, Amarin, Amgen, AstraZeneca, Bayer, Beren, Boehringer Ingelheim, Boston Scientific, Bristol-Myers Squibb, Cardax, CellProthera, Cereno Scientific, Chiesi, CSL Behring, Eisai, Ethicon, Faraday Pharmaceuticals, Ferring Pharmaceuticals, Forest Laboratories, Fractyl, Garmin, HLS Therapeutics, Idorsia, Ironwood, Ischemix, Janssen, Javelin, Lexicon, Lilly, Medtronic, Merck, Moderna, MyoKardia, NirvaMed, Novartis, Novo Nordisk, Owkin, Pfizer, PhaseBio, PLx Pharma, Recardio, Regeneron, Reid Hoffman Foundation, Roche, Sanofi, Stasys, Synaptic, The Medicines Company, 89Bio; Royalties: Elsevier (Editor, Braunwald’s Heart Disease); Site Co-Investigator: Abbott, Biotronik, Boston Scientific, CSI, Endotronix, St. Jude Medical (now Abbott), Philips, Svelte; Trustee: American College of Cardiology; Unfunded Research: FlowCo, Takeda. John Spertus provides consultative services for Bayer, Merck, Janssen, Novartis, Bristol Meyers Squibb, United Healthcare, and Terumo; Has research grants from the American College of Cardiology Foundation, Abbott Vascular, Bristol Meyers Squibb and Jannsen; Owns the copyright to the SAQ, KCCQ, and PAQ; and serves on the Board of Directors for Blue Cross/Blue Shield of Kansas City. Gregg W. Stone has received speaker honoraria from Medtronic, Pulnovo, Infraredx; has served as a consultant to Valfix, TherOx, Robocath, HeartFlow, Ablative Solutions, Vectorious, Miracor, Neovasc, Abiomed, Ancora, Elucid Bio, Occlutech, CorFlow, Apollo Therapeutics, Impulse Dynamics, Vascular Dynamics, Shockwave, V-Wave, Cardiomech, Gore, Amgen; and has equity/options from Ancora, Cagent, Applied Therapeutics, Biostar family of funds, SpectraWave, Orchestra Biomed, Aria, Cardiac Success, Valfix, Xenter. Stone’s daughter is an employee at Medtronic. Institutional disclosure: Stone’s employer, Mount Sinai Hospital, receives research support from Abbott, Abiomed, Bioventrix, Cardiovascular Systems Inc, Phillips, Biosense-Webster, Shockwave, Vascular Dynamics and V-wave. All other authors declared no potential conflicts of interest with respect to the research, authorship, and/or publication of this article.
