# Supplementary material for: Correlation Between Periprocedural Myocardial Infarction, Mortality, and Quality of Life in Coronary Revascularization Trials: A Meta-analysis
Source: J Soc Cardiovasc Angiogr Interv. 2023 Apr 3;2(3):100591. doi: 10.1016/j.jscai.2023.100591 (PMC11307952; doi:10.1016/j.jscai.2023.100591)
Supplement: Supplementary Material [file mmc1.docx]

**APPENDIX**

| Full Search Strategy |
| --- |
| Supplementary Table 1. Details of the definitions of periprocedural myocardial infarction used in the included trials. |
| Supplementary Table 2. Details of Patient Characteristics. |
| Supplementary Table 3. Procedural Characteristics. |
| Supplementary Table 4. Details of Medical Therapy. |
| Supplementary Table 5. Cochrane Collaboration’s tool for assessing the risk of bias for the included trials. |
| Supplementary Table 6. Subgroup analyses of the correlations between periprocedural myocardial infarction (pMI) and all-cause and cardiac mortality. |
| Supplementary Table 7. Sensitivity analyses of the impact of study-level factors on the correlation between periprocedural myocardial infarction (pMI) and all-cause and cardiac mortality. |
| Supplementary Figure 1. Preferred Reporting Items for Systematic Reviews and Meta-Analyses (PRISMA) flow diagram. |
| Supplementary Figure 2. Subgroup analysis of the trials that required a raise in cardiac biomarkers (CK-MB or Troponin) >5 times the upper reference limits to diagnose periprocedural myocardial infarction (pMI) for the association between the relative risk (RR) for the surrogate end point of periprocedural myocardial infarction and the incidence rate ratio (IRR) for the true end points of all-cause or cardiac mortality. |
| Supplementary Figure 3. Subgroup analysis of the trials that required a raise in cardiac biomarkers (CK-MB or Troponin) ≤5 times the upper reference limits to diagnose periprocedural myocardial infarction (pMI) for the association between the relative risk (RR) for the surrogate end point of periprocedural myocardial infarction and the incidence rate ratio (IRR) for the true end points of all-cause or cardiac mortality. |
| Supplementary Figure 4. Subgroup analysis of the trials that required a rise in CK-MB >5 times the upper reference limits to diagnose periprocedural myocardial infarction (pMI) for the association between the relative risk (RR) for the surrogate endpoint of pMI and the incidence rate ratio (IRR) of the true endpoint for all-cause or cardiac mortality. |
| Supplementary Figure 5. Subgroup analysis of the trials that required a rise in CK-MB ≤5 times the upper reference limits to diagnose periprocedural myocardial infarction (pMI) for the association between the relative risk (RR) for the surrogate endpoint of pMI and the incidence rate ratio (IRR) of the true endpoint for all-cause or cardiac mortality. |
| Supplementary Figure 6. Subgroup analysis of the trials that included only patients with multivessel disease for the association between the relative risk (RR) for the surrogate endpoint of periprocedural myocardial infarction (pMI) and the incidence rate ratio (IRR) of the true endpoint for all-cause or cardiac mortality. |
| Supplementary Figure 7. Subgroup analysis of the trials that included only patients with left main disease for the association between the relative risk (RR) for the surrogate endpoint of periprocedural myocardial infarction (pMI) and the incidence rate ratio (IRR) of the true endpoint for all-cause or cardiac mortality. |
| Supplementary Figure 8. Subgroup analysis of the trials where only drug eluting stents (DES) were used for the association between the relative risk (RR) for the surrogate endpoint of periprocedural myocardial infarction (pMI) and the incidence rate ratio (IRR) of the true endpoint for all-cause or cardiac mortality. |
| Supplementary Figure 9. Subgroup analysis of the trials that were initiated after year 2000 for the association between the relative risk (RR) for the surrogate endpoint of periprocedural myocardial infarction (pMI) and the incidence rate ratio (IRR) of the true endpoint of all-cause or cardiac mortality. |
| Supplementary Figure 10. Subgroup analysis of the trials with follow-up duration >1 year for the association between the relative risk (RR) for the surrogate endpoint of periprocedural myocardial infarction (pMI) and the incidence rate ratio (IRR) for true endpoint of all-cause or cardiac mortality. |
| Supplementary Figure 11. Subgroup analysis of the trials with follow-up duration ≤1 year for the association between the relative risk (RR) for the surrogate endpoint of periprocedural myocardial infarction (pMI) and the incidence rate ratio (IRR) for true endpoint of all-cause or cardiac mortality. |
| Supplementary Figure 12. Subgroup analysis of the trials with follow-up duration >4 year for the association between the relative risk (RR) for the surrogate endpoint of periprocedural myocardial infarction (pMI) and the incidence rate ratio (IRR) for true endpoint of all-cause or cardiac mortality. |
| Supplementary Figure 13. Subgroup analysis of the trials with follow-up duration ≤4 year for the association between the relative risk (RR) for the surrogate endpoint of periprocedural myocardial infarction (pMI) and the incidence rate ratio (IRR) for true endpoint of all-cause or cardiac mortality. |
| Supplementary Figure 14. Correlation between the logarithm of the rate of the surrogate endpoint of periprocedural myocardial infarction (pMI) and the incidence rate (IR) for the true endpoint of all-cause mortality or cardiac mortality in PCI. |
| Supplementary Figure 15. Correlation between the logarithm of the rate of the surrogate endpoint of periprocedural myocardial infarction (pMI) and the incidence rate (IR) for the true endpoint of all-cause mortality or cardiac mortality in CABG. |
| Supplementary References |

**Full Search Strategy**

Ovid MEDLINE® ALL - 1946 to January 26, 2022

Searched on January 26, 2022

Limited to RCTs via BMJ's study design search filter available from: https://bestpractice.bmj.com/info/toolkit/learn-ebm/study-design-search-filters/

Line # | Search

1 Percutaneous Coronary Intervention/

2 (percutaneous coronary intervention* or percutaneous coronary revascularization* or PCI or percutaneous coronary angioplasty or stent or stents or stenting).tw.

3 Angioplasty, Balloon, Coronary/

4 (coronary balloon angioplasties or coronary balloon angioplasty or transluminal coronary balloon dilation or coronary artery balloon dilation or percutaneous transluminal coronary angioplasty or coronary angioplasty or coronary angioplasties or PTCA).tw.

5 or/1-4

6 Coronary Artery Bypass/

7 (coronary adj2 (bypass or graft)).tw.

8 (CABG or aortocoronary anastomosis or total arterial revascularization or total arterial revascularisation or Multiple arterial revascularization or multiple arterial revascularisation).tw.

9 Coronary Artery Bypass, Off-Pump/

10 Internal Mammary-Coronary Artery Anastomosis/

11 ((Right Internal Mammary Artery or RIMA or Coronary Internal Mammary Artery or arteria mammaria interna or arteria thoracica interna or internal thoracic artery or mammary internal artery) and (transplant* or graft* or anastomosis)).tw.

12 (surgical revascularization or cardiac muscle revascularisation or cardiac muscle revascularization or coronary revascularisation or coronary revascularization or heart muscle revascularisation or heart myocardium revascularisation or heart revascularisation or heart revascularization or internal mammary arterial anastomosis or internal mammary arterial implantation or internal mammary artery anastomosis or internal mammary artery graft or internal mammary artery implant or internal mammary artery implantation or internal mammary-coronary artery anastomosis or myocardial revascularisation or myocardial revascularization or myocardium revascularisation or myocardium revascularization or transmyocardial laser revascularisation or transmyocardial laser revascularization or vineberg operation).tw.

13 or/6-12

14 "randomized controlled trial".pt.

15 (random$ or placebo$ or single blind$ or double blind$ or triple blind$).ti,ab

16 (retraction of publication or retracted publication).pt.

17 or/14-16

18 (animals not humans).sh.

19 ((comment or editorial or meta-analysis or practice-guideline or review or letter) not "randomized controlled trial").pt.

20 (random sampl$ or random digit$ or random effect$ or random survey or random regression).ti,ab. not "randomized controlled trial".pt.

21 17 not (18 or 19 or 20)

22 5 and 13

23 22 and 21

**Supplementary Table 1. Details of the definitions of periprocedural myocardial infarction used in the included trials.**

| Trial | Type of definition used | Details of the definition/source |
| --- | --- | --- |
| BARI^1^ | Protocol definition | *Postprocedure MI was defined by the presence of a new, two-grade (Minnesota Code) worsening in the Q-wave on the postprocedure versus the preprocedure ECG. According to protocol, cardiac enzymes were not used to define MI within 96 hours of a revascularization procedure because of the recognized difficulties in comparing the clinical significance of cardiac enzyme elevations after CABG compared with after PCI.* |
| Blazek et al. (2013)^2^ | First Universal Definition of MI | *From: Thygesen K, Alpert JS, White HD, et al., Joint ESC/ACCF/AHA/WHF task force for the redefinition of myocardial infarction. J Am Coll Cardiol 2007;50:2173–95* |
| Boudriot et al.^3^ | Protocol definition | *MI was defined as an increase in CK-MB activity >3 times the URL after PCI and >5 times after CABG. In addition, standard ECG criteria were applied. Outcomes were considered within 30 days of the procedure.* |
| CARDia^4^ | Protocol definition | *During the first 7 days following revascularization when any of the following criteria are fulfilled:*  *- Development of new abnormal Q waves not present on the baseline ECG*  *- Pathological Q wave defined as >1mm (0.04 s) duration and >2mm (0.08 s) deep or having a depth > one-fourth of the corresponding R wave amplitude in ≥ 2 contiguous leads.*  *- Enzyme changes defined as a Ck rise to >3 times the URL, with elevation in CK-MB/CK ratio >10%.* |
| EXCEL^5^ | Protocol definition | *Defined as the occurrence within 72 hours of either PCI or CABG. Includes:*  *- CK-MB >10x URL, OR*  *- CK-MB >5x URL, PLUS*   - *New pathological Q waves in at least 2 contiguous leads or new persistent non-rate related LBBB, or* - *Angiographically documented graft or native coronary artery occlusion or new severe stenosis with thrombosis and/or diminished epicardial flow, or* - *Imaging evidence of new loss of viable myocardium or new regional wall motion abnormality* |
| FAME 3^6^ | Protocol definition | *In both groups, the biomarker threshold used to define a procedural MI was any elevation of the cardiac troponin level to more than 10 times the 99th percentile of the URL within 72 hours after the procedure in patients who had normal levels at baseline or an increase in the cardiac troponin level of more than 20% in patients who had elevated levels at baseline. In addition, at least one of the following criteria needed to be met: new pathologic Q waves or new LBBB, angiographic documentation of new graft or major native coronary occlusion, or imaging evidence of new loss of viable myocardium or a new regional wall-motion abnormality.* |
| FREEDOM^7,8^ | Protocol definition | *In the first 30 days, MI is defined as the presence of new Q waves in ≥2 contiguous leads at the 30-day ECG when compared with baseline ECG.* |
| Hong et al.^9^ | Not reported definition | **-** |
| NOBLE^10^ | Protocol definition | *Adapted to match the definition of procedural MI applied in the EXCEL trial.*  *Defined as the occurrence within 72 hours of either PCI or CABG. Includes:*  *- CK-MB >10x URL, OR*  *- CK-MB >5x URL, PLUS*   - *New pathological Q waves in at least 2 contiguous leads or new persistent non-rate related LBBB, or* - *Angiographically documented graft or native coronary artery occlusion or new severe stenosis with thrombosis and/or diminished epicardial flow, or* - *Imaging evidence of new loss of viable myocardium or new regional wall motion abnormality* |
| SYNTAX^11,12^ | Protocol definition | *Within the first 7 days post intervention (PCI or CABG).*  *Includes:*  *- Either new, abnormal Q waves and 1 ratio of peak CK–MB/CK >10%, or*  *- New, abnormal Q-waves and 1 plasma level of CK-MB >5x URL* |
| Blazek et al. (2015)^13^ | First Universal Definition of MI | *From: Thygesen K, Alpert JS, White HD, et al., Joint ESC/ACCF/AHA/WHF task force for the redefinition of myocardial infarction. J Am Coll Cardiol 2007;50:2173–95* |
| VA CARDS^14^ | Protocol definition | *A 5-fold increase in CK-MB plus diagnostic new Q waves on ECG. No details on time after procedure are provided.* |

CABG, coronary artery bypass grafting; CK, creatine kinase; CK-MB, creatine kinase myocardial band; ECG, electrocardiogram; LBBB, left bundle branch block; MI, myocardial infarction; PCI, percutaneous coronary intervention; URL, upper reference limit.

**Supplementary Table 2. Details of Patient Characteristics (continues).**

| Trial | Treatment | Age, mean (SD), median [IQR] | Female (%) | BMI (SD) [IQR] | Smoking (%) | DM (%) | Insulin (%) | CAD, family history (%) | Statin (%) | HTN (%) | HCL/ HLD (%) | PVD (%) | Carotid artery disease (%) | Prior Stroke (%) | Prior MI (%) | Prior TIA (%) | Prior CHF (%) | Prior PCI (%) | Prior CABG (%) | LVEF (SD) [IQR] | SA (%) | UA (%) | ACS (%) |
| --- | --- | --- | --- | --- | --- | --- | --- | --- | --- | --- | --- | --- | --- | --- | --- | --- | --- | --- | --- | --- | --- | --- | --- |
| BARI^1^ | PCI | 39% above 65 | 27 | 28 | 72 | 19 | 9 | - | - | 49 | - | 17 | - | - | 54 | - | 9 | - | - | 78% normal LVEF | 32 | 68 | - |
|  | CABG | 39% above 65 | 26 | 30 | 69 | 20 | 9 | - | - | 49 | - | 16 | - | - | 55 | - | 9 | - | - | 80% normal LVEF | 30 | 70 | - |
| Blazek et al. (2013)^2^ | PCI | 62.5 (10.2) | 28 | 28.2 (3.8) | 25 | 34 | - | 18 | - | 72 | 70 | - | - | - | 45 | - | - | 0 | 0 | 62 (15) | - | - | - |
|  | CABG | 61.6 (10.0) | 15 | 27.2 (3.4) | 25 | 25 | - | 17 | - | 71 | 73 | - | - | - | 45 | - | - | 0 | 0 | 63 (11) | - | - | - |
| Boudriot et al.^3^ | PCI | 66 [62-73] | 28 | 27.2 [24.6-31.5] | - | 40 | - | - | - | 82 | 68 | - | - | 3 | 19 | - | - | - | - | 65.0 [55.0-70.0] | - | - | - |
|  | CABG | 69 [63-73] | 22 | 27.0 [24.9-30.1] | - | 33 | - | - | - | 82 | 64 | - | - | 6 | 14 | - | - | - | - | 65.0 [55.0-68.0] | - | - | - |
| CARDia^4^ | PCI | 64.3 (8.5) | 29.3 | 29.2 (4.9) | 29.3 | 100 | 36.5 | - | - | 76.6 | 92.9 | 2.4 | - | - | - | - | - | - | - | - | - | - | - |
|  | CABG | 63.6 (9.1) | 22.1 | 29.4 (5.3) | 29.1 | 100 | 39.1 | - | - | 80.6 | 87.3 | 5.2 | - | - | - | - | - | - | - | - | - | - | - |
| EXCEL^5^ | PCI | 66.0 (9.6) | 23.8 | 28.6 (5.0) | 23.4 | 30.2 | 7.7 | - | - | 74.2 | 70.5 | 10.2 | - | - | 17.8 | 5.5 | 7.1 | 18.4 | 0 | 57.0 (9.6) | 52.7 | 24.1 | - |
|  | CABG | 65.9 (9.5) | 22.5 | 28.8 (4.9) | 20.2 | 28.0 | 7.7 | - | - | 73.2 | 68.1 | 8.8 | - | - | 16.8 | 7.0 | 6.2 | 15.9 | 0 | 57.3 (9.0) | 52.8 | 24.5 | - |
| FAME 3^6^ | PCI | 65.2 (8.6) | 18.6 | 28.6 (4.5) | 19.2 | 28.3 | 7.3 | 32.5 | - | 71.2 | 68.9 | - | - | 6.5 | 33.3 | - | - | 13 | - | LVEF <50% in 18.2% | - | - | - |
|  | CABG | 65.1 (8.3) | 16.7 | 28.7 (4.3) | 18.4 | 28.8 | 8.2 | 28.8 | - | 75.0 | 71.7 | - | - | 7.6 | 33.5 | - | - | 14 | - | LVEF <50% in 17.6% | - | - | - |
| FREEDOM^7,8^ | PCI | 63.2 (8.9) | 26.8 | 29.6 (5.4) | 14.8 | 100 | 33.8 | - | 82.1 | 84.6 | - | - | - | 3.9 | 26.2 | - | - | - | - | 65.7 (12.1) | - | - | 31.9 |
|  | CABG | 63.1 (9.2) | 30.5 | 29.8 (5.3) | 16.6 | 100 | 30.9 | - | 82.6 | 85.1 | - | - | - | 3.0 | 25.0 | - | - | - | - | 66.6 (10.5) | - | - | 29.5 |
| Hong et al.^9^ | PCI | 60.5 (9.6) | 36.1 | 25.5 (2.9) | 40.3 | 37.0 | - | 9.3 | - | 50.4 | 54.6 | - | - | 2.5 | 21.8 | - | - | 0 | 0 | 52.8 (8.8) | - | 50.4 | - |
|  | CABG | 61.4 (9.9) | 35.7 | 26.6 (3.9) | 45.7 | 48.6 | - | 10.0 | - | 55.7 | 51.4 | - | - | 2.9 | 22.9 | - | - | 0 | 0 | 51.9 (9.1) | - | 42.9 | - |
| NOBLE^10^ | PCI | 66.2 (9.9) | 20.0 | 27.9 (4.5) | 19 | 15 | - | 58 | 82 | 65.2 | - | - | - | - | - | - | - | 19.6 | 0.7 | 60 [55-65] | 82.1 | - | 17.9 |
|  | CABG | 66.2 (9.4) | 24.0 | 28.1 (4.4) | 22 | 15 | - | 56 | 78 | 65.7 | - | - | - | - | - | - | - | 19.9 | 0.3 | 60 [52-64] | 82.9 | - | 16.9 |
| SYNTAX^11,12^ | PCI | 65.2 (9.7) | 23.6 | 28.1 (4.8) | 18.5 | 25.6 | 24.6 | - | - | 68.9 | 78.7 | - | 8.1 | 3.9 | 31.9 | 4.3 | 4.0 | - | - | - | 56.9 | 28.9 | - |
|  | CABG | 65.0 (9.8) | 21.1 | 27.9 (4.5) | 22.0 | 24.6 | 9.9 | - | - | 64.0 | 77.2 | - | 8.4 | 4.8 | 33.8 | 5.2 | 5.3 | - | - | - | 57.2 | 28.0 | - |
| Blazek et al. (2015) ^13^ | PCI | 66 (59-72) | 31 | 28.0 (3.7) | 14 | 28 | - | - | - | 83 | 55 | - | - | 3 | 23 | - | - | 0 | 0 | 65 [60-66] | - | - | - |
|  | CABG | 66 (59-71) | 29 | 26.9 (4.0) | 18 | 25 | - | - | - | 85 | 55 | - | - | 9 | 23 | - | - | 0 | 0 | 65 [60-70] | - | - | - |
| VA CARDS^14^ | PCI | 62.7 (7.1) | 1 | 32.8 (5.7) | 27.7 | 100 | 47.5 | - | 90.1 | 96 | - | 10.9 | - | 6.9 | 45.5 | - | - | 34.7 | 3 | LVEF <55% in 51.1% | - | - | 9.9 |
|  | CABG | 62.1 (7.4) | 1 | 22 (5.7) | 20.6 | 100 | 47.9 | - | 85.1 | 95.7 | - | 17 | - | 8.5 | 37.2 | - | - | 20.2 | 1.1 | LVEF <55% in 36.4% | - | - | 8.5 |

**Supplementary Table 2. Details of Patient Characteristics (continued).**

| Trial | Treatment | Bifurcation (%) | Bifurcation or trifurcation of the distal left artery (%) | Diseased non-left main coronary arteries (0,1,2,3) (%) | NYHA Class I (%) | NYHA Class II (%) | NYHA Class III (%) | NYHA Class IV (%) | EuroSCORE (SD) [IQR] | SYNTAX score (SD) [IQR] |
| --- | --- | --- | --- | --- | --- | --- | --- | --- | --- | --- |
| BARI^1^ | PCI | - | - | - | - | - | - | - | - | - |
|  | CABG | - | - | - | - | - | - | - | - | - |
| Blazek et al. (2013)^2^ | PCI | - | - | - | - | - | - | - | - | - |
|  | CABG | - | - | - | - | - | - | - | - | - |
| Boudriot et al.^3^ | PCI | - | - | (28,35,26,11) | - | - | - | - | - | 24.0 [19.0-29.0] |
|  | CABG | - | - | (29,27,28,17) | - | - | - | - | - | 23.0 [14.8-29.0] |
| CARDia^4^ | PCI | - | - | - | - | - | - | - | - | - |
|  | CABG | - | - | - | - | - | - | - | - | - |
| EXCEL^5^ | PCI | - | 81.3 | (17.2,30.8,34.3,17.) | - | - | - | - | - | 32.2% (<22); 42.8% (23-32); 25.1% (>33) |
|  | CABG | - | 77.4 | (17.5,30.5,30.8,19.0) | - | - | - | - | - | 39.3% (<22); 37.3% (23-32); 23.4% (>33) |
| FAME 3^6^ | PCI | 69.1 | - | - | - | - | - | - | - | 26 (7.1) |
|  | CABG | 66.4 | - | - | - | - | - | - | - | 25.8 (7.1) |
| FREEDOM^7,8^ | PCI | - | - | - | - | - | - | - | 2.7 (2.4) | 26.2 (8.4) |
|  | CABG | - | - | - | - | - | - | - | 2.8 (2.5) | 26.1 (8.8) |
| Hong et al.^9^ | PCI | - | - | - | - | - | - | - | - | - |
|  | CABG | - | - | - | - | - | - | - | - | - |
| NOBLE^10^ | PCI | - | - | - | 53 | 29.6 | 13 | 5 | 2 [2-4] | 22.5 (7.5) |
|  | CABG | - | - | - | 43 | 33.0 | 17 | 7 | 2 [2-4] | 22.4 (8.0) |
| SYNTAX^11,12^ | PCI | 72.4 | - | - | - | - | - | - | 3.8 (2.6) | 28.4 (11.5) |
|  | CABG | 73.3 | - | - | - | - | - | - | 3.8 (2.7) | 29.1 (11.4) |
| Blazek et al. (2015)^13^ | PCI | - | - | - | - | - | - | - | - | - |
|  | CABG | - | - | - | - | - | - | - | - | - |
| VA CARDS^14^ | PCI | - | - | - | - | - | - | - | - | 21.5 (8.9) |
|  | CABG | - | - | - | - | - | - | - | - | 22.7 (10.6) |

ACS, acute coronary syndrome; BMI, body mass index; CABG, coronary artery bypass grafting; CAD, coronary artery disease; CHF, chronic heart failure; DM, diabetes mellitus; HTN, hypertension; HCL, hypercholesterolemia; HLD, hyperlipidemia; LVEF, left ventricular ejection fraction; MI, myocardial infarction; NYHA, New York Heart Association; PCI, percutaneous coronary intervention; PVD, peripheral vascular disease; SA, stable angina pectoris; TIA, transient ischemic attack; UA, unstable angina.

**Supplementary Table 3. Procedural Characteristics (continued).**

| Trial | Treatment | Aspirin (%) | Thienophyridine (%) | Ticagrelor (%) | GP Inhibitor (%) | Statin (%) | Beta-blocker (%) | ACEI or ARB (%) | Calcium channel blocker (%) | No. of lesions (SD or IQR) | CR (%) | No. of stents (SD) [IQR] | DES use (%) | Type of stent | Total stent length, mm (SD) | Stent diameter, mm (SD) [IQR] | No. of non-LMCA stents (0,1,2, bifurcation) (%) | Bifurcation technique (1 stent, 2 stent) (%) | Intravascular ultrasound, (any, pre-PCI, post-PCI) (%) |
| --- | --- | --- | --- | --- | --- | --- | --- | --- | --- | --- | --- | --- | --- | --- | --- | --- | --- | --- | --- |
| BARI^1^ | PCI | 84 | - | - | - | 65 | - | - | - | - | - | - | 0 | BMS | - | - | - | - | - |
|  | CABG | 86 | - | - | - | 63 | - | - | - | - | - | - | - | - | - | - | - | - | - |
| Blazek et al. (2013)^2^ | PCI | - | - | - | - | - | - | - | - | - | - | 1.2 (0.4) | 0 | BMS | 15.1 (4.3) | - | - | - | - |
|  | CABG | - | - | - | - | - | - | - | - | - | - | - | - | - | - | - | - | - | - |
| Boudriot et al.^3^ | PCI | - | - | - | - | 97 | 99 | 98 | - | - | - | - | 100 | DES | - | - | - | - | - |
|  | CABG | - | - | - | - | 94 | 95 | 92 | - | - | - | - | - | - | - | - | - | - | - |
| CARDia^4^ | PCI | - | - | - | - | - | - | - | - | 3.6 | - | - | 69 | BMS, DES | 71 | - | - | - | - |
|  | CABG | - | - | - | - | - | - | - | - | - | - | - | - |  | - | - | - | - | - |
| EXCEL^5^ | PCI | 95.9 | 95.9 | 6.9 | - | 94.7 | 81.8 | 55.7 | 5.8 | 1.9 (1.1) | - | 2.4 (1.5) | 100 | DES | 49.1 (35.6) | - | - | - | (76.2,-,-) |
|  | CABG | 92.1 | 31.0 | 0.2 | - | 88.0 | 88.1 | 40.1 | 6.8 | 2.6 (0.8) | - | - | - | - | - | - | - | - | - |
| FAME 3^6^ | PCI | - | - | - | - | - | - | - | - | 4.3 (1.3) | - | 3.7 (1.9) | 100 | DES | 80 (52-116) | - | - | - | 11.7 |
|  | CABG | - | - | - | - | - | - | - | - | 4.2 (1.2) | - | - | - | - | - | - | - | - | - |
| FREEDOM^7,8^ | PCI | 98.4 | 97.8 | - | - | 83.7 | 79.3 | 80.2 | 24.7 | 5.7 (2.2) | - | 3.5 (1.4) | 100 | DES | 26.1 (14.2) | - | - | - | - |
|  | CABG | 85.9 | 23.9 | - | - | 81.1 | 76.1 | 60.2 | 18.0 | 5.7 (2.2) | - | - | - | - | - | - | - | - | - |
| Hong et al.^9^ | PCI | - | - | - | - | - | - | - | - | - | - | 1.2 (0.2) | 100 | DES | 22.6 (4.8) | 2.9 (0.3) | - | - | - |
|  | CABG | - | - | - | - | - | - | - | - | - | - | - |  | - |  | - | - | - | - |
| NOBLE^10^ | PCI | 91.0 | 95.6 | - | 18.6 | - | - | - | - | 2 (1-3) | 91.7 | - | 100 | DES | - | 4.0 [4.0-4.5] | (52.7, 32.3, 9.3, 85.8) | (-, 29.7) | (-,45.6,72.6) |
|  | CABG | - | - | - | - | - | - | - | - | 2 (2-3) | - | - | - | - | - | - | - | - |  |
| SYNTAX^11,12^ | PCI | 96.3 | 96.8 | 1.9 | - | 86.7 | 81.3 | 78.6 | 25.8 | 4.3 (1.8) | 61.3 | 4.6 (2.3) | 100 | DES | 86.1 (47.9) | - | - | - | - |
|  | CABG | 88.5 | 19.5 | 4.8 | - | 74.5 | 78.6 | 68.4 | 18.4 | 4.4 (1.8) | 56.3 | - | - | - | - | - | - | - | - |
| Blazek et al. (2015)^13^ | PCI | 100 | 100 | - | - | 99 | 99 | 100 | - | - | - | - | 95.4 | DES, BMS | - | - | - | - | - |
|  | CABG | 100 | 34 | - | - | 97 | 97 | 97 | - | - | - | - | - | - | - | - | - | - | - |
| VA CARDS^14^ | PCI | - | - | - | - | - | - | - | - | - | - | - | 100 | DES | - | - | - | - | - |
|  | CABG | - | - | - | - | - | - | - | - | - | - | - | - | - | - | - | - | - | - |

**Supplementary Table 3. Procedural Characteristics (continued).**

| Trial | Treatment | LIMA (%) | BIMA (%) | OPCAB (%) | LIMA+ SV grafting (%) | No. of grafts, mean (SD) | No. of arterial grafts, mean (SD) | No. of venous grafts, mean (SD) | No. of grafts, (1,2,3,4,5) (%) | Ultrasound (epi-aortic or transesophageal-aortic, epi-aortic, transesophageal) |
| --- | --- | --- | --- | --- | --- | --- | --- | --- | --- | --- |
| BARI^1^ | PCI | - | - | - | - | - | - | - | - | - |
|  | CABG | - | - | - | - | - | - | - | - | - |
| Blazek et al. (2013)^2^ | PCI | - | - | - | - | - | - | - | - | - |
|  | CABG | - | - | - | - | - | - | - | - | - |
| Boudriot et al.^3^ | PCI | - | - | - | - | - | - | - | - | - |
|  | CABG | 99.0 | - | - | - | - | - | - | - | - |
| CARDia^4^ | PCI | - | - | - | - | - | - | - | - | - |
|  | CABG | 94 | - | - | - | 2.9 | - | - | - | - |
| EXCEL^5^ | PCI | - | - | - | - | - | - | - | - | - |
|  | CABG | 94.9 | 27.7 | 28.3 | - | 2.6 (0.8) | 1.4 (0.6) | 1.2 (0.9) | - | (43.6, 12.6, 40.8) |
| FAME 3^6^ | PCI | - | - | - | - | - | - | - | - | - |
|  | CABG | 97 | - | 24.1 | - | 3.4 (1) | - | - | - | - |
| FREEDOM^7,8^ | PCI | - | - | - | - | - | - | - | - | - |
|  | CABG | 89.5 | - | 17.4 | - | 2.9 (0.8) | - | - | - | - |
| Hong et al.^9^ | PCI | - | - | - | - | - | - | - | - | - |
|  | CABG | - | - | - | - | - | - | - | - | - |
| NOBLE^10^ | PCI | - | - | - | - | - | - | - | - | - |
|  | PCI | - | - | - | - | - | - | - | - | - |
| SYNTAX^11,12^ | PCI | - | - | - | - | - | - | - | - | - |
|  | CABG | - | 24.1 | 14.3 | - | 2.8 (0.7) | - | - | - | - |
| Blazek et al. (2015)^13^ | PCI | - | - | - | - | - | - | - | - | - |
|  | CABG | 98.5 | - | 95.8 | - | - | - | - | - | - |
| VA CARDS^14^ | PCI | - | - | - | - | - | - | - | - | - |
|  | CABG | - | - | - | - | - | - | - | - | - |

ACEI, angiotensin-converting enzyme inhibitor; ARB, angiotensin II receptor blockers; BIMA, bilateral internal mammary artery; BMS, bare-metal stent; CR, complete revascularization; DES, drug-eluting stent; GP, glycoprotein IIb/IIIa; LIMA, left internal mammary artery; LMCA, left main coronary artery; OPCAB, off-pump coronary artery bypass grafting; SV, Saphenous vein.

**Supplementary Table 4. Details of Medical Therapy.**

| Trial | Description of Medical Therapy |
| --- | --- |
| BARI^1^ | Aspirin (PCI: 84%, CABG: 86%)  Beta blockers, calcium antagonists, or long-acting nitrates (PCI: 75%, CABG: 66%)  Statin (PCI: 65%, CABG 63%) |
| Blazek et al. (2013)^2^ | - PCI: aspirin (100 mg/day, indefinitely); ticlopidine or clopidogrel (4 weeks, following a loading dose the day before the procedure) - CABG: aspirin (100 mg/day, indefinitely)   Beta-blocker (PCI: 74%, CABG: 75%)  ACE inhibitor/AT-1 antagonist: (PCI: 73%, CABG: 71%)  Statin (PCI: 68%, CABG: 68%)  Aspirin (PCI: 74%, CABG: 69%)  Thienopyridines (PCI: 10%, CABG: 8%)  Nitrates (PCI: 20%, CABG: 19%)  Calcium antagonists (PCI: 22%, CABG: 15%)  Antidiabetic medication (PCI: 21%, CABG: 18%) |
| Boudriot et al.^3^ | - PCI: antiplatelet therapy (>100 mg/day, indefinitely); clopidogrel (75 mg/day, ≥12 months); Glycoprotein IIb/IIIa inhibitor use was left to the discretion of the operator. - CABG: aspirin (100 mg/day, indefinitely) - BOTH: other pharmacological treatments such as statins, angiotensin-converting enzyme inhibitors, and betablockers were recommended based on current practice in both treatment groups.   At discharge:   - Aspirin (PCI: 100%, CABG: 100%) - Clopidogrel (PCI: 100, CABG: 32%) - Beta-blocker (PCI: 99, CABG: 95%) - ACE inhibitor/AT-1 antagonist (PCI: 98%, CABG: 92%) - Statins (PCI: 97%, CABG: 94%) - Routine administration of abciximab and clopidogrel for 1 to 3 months after BMS placement or 12 months after DES placement. |
| CARDia^4^ | Aspirin (PCI: 83.4%, CABG: 87.2%)  Clopidogrel (PCI: 54.4%, CABG: 10.3%)  Aspirin and clopidogrel (PCI: 50.9%, CABG: 16.5%)  Statins (PCI: 83.4%, CABG: 89.3%)  ACE inhibitors: (PCI: 56.1%, CABG: 60.3%)  Oral hypoglycemics (PCI: 65.5%, CABG: 61.1%)  Insulin (PCI: 29.8%, CABG: 40.9%) |
| EXCEL^5^ | Aspirin (PCI: 93.0%, CABG: 93.6%)  P2Y12 receptor inhibitor (PCI: 61.6%, CABG: 21.0%)  Clopidogrel or ticlopidine (PCI: 50.0%, CABG: 20.3%)  Clopidogrel (PCI: 50.0%, CABG: 20.2%)  Ticlopidine (PCI: 0.0%, CABG: 0.1%)  Prasugrel or ticagrelor (PCI: 11.6%, CABG: 0.8%)  Prasugrel (PCI: 8.5%, CABG: 0.4%)  Ticagrelor (PCI: 3.1%, CABG: 0.4%)  Beta-blockers (PCI: 86.6%, CABG: 94.3%)  Calcium channel blockers (PCI: 18.3%, CABG: 19.1%)  ACE inhibitors or receptor blockers (PCI: 66.7%, CABG: 59.4%)  Aldosterone antagonist: (PCI: 1.6%, CABG: 1.7%)  Diuretic: (PCI: 17.1%, CABG: 38.8%)  Anti-arrhythmic agent: (PCI: 3.1%, CABG: 17.4%)  Statins: (PCI: 97.5%, CABG: 96.2%)  Chronic oral anticoagulant (PCI: 5.2%, CABG: 10.8%) |
| FAME 3^6^ | All the patients in both groups were to receive aspirin and a high-dose statin, as well as guideline-directed medical therapy. Patients undergoing PCI were to receive a second antiplatelet medication for at least 6 months after PCI. |
| FREEDOM^7,8^ | Aspirin (PCI: 95.3%, CABG: 95.4%)  Thienopyridine (PCI: 58.7%, CABG: 22.8%)  Warfarin (PCI: 1.4%, CABG: 1.7%)  Statin (PCI: 91.4%, CABG: 89.9%)  Beta blocker (PCI: 82.6%, 82.8%)  ACE inhibitor (PCI: 67.4%, 66.7%)  Angiotensin-II receptor antagonist (PCI: 31.6%, CABG: 29.4%)  Calcium-channel blocker (PCI: 28.4%, CABG: 24.8%)  H2-receptor blocker (PCI: 19.7%, CABG: 20.8%) |
| Hong et al.^9^ | PCI: aspirin (indefinitely); clopidogrel or ticlopidine (6 months) |
| NOBLE^10^ | - All: aspirin (75–150 mg/day, indefinitely); clopidogrel (75 mg/day, 12 months if acute coronary syndrome is present) - PCI: clopidogrel (75 mg/day, 12 months); prasugrel or ticagrelor could be substituted for clopidogrel at the discretion of the PCI operator. |
| SYNTAX^11,12^ | Acetylsalicylic acid (Aspirin) (PCI: 87.1%, CABG: 85.0%)  Thienopyridine (PCI: 32.0%, CABG: 12.1%)  Other antiplatelet (PCI: 4.1%, CABG: 3.3%)  Acetylsalicylic acid (Aspirin) and Antiplatelet (PCI: 27.4%, CABG: 9.1%) |
| Blazek et al. (2015)^13^ | PCI: aspirin (100 mg/day, indefinitely); clopidogrel (75 mg/day, ≥12 months)  CABG: aspirin (100 mg/day, indefinitely) |
| VA CARDS^14^ | None reported |

ACE, angiotensin converting enzyme; CABG, coronary artery bypass grafting; IU, international units; PCI, percutaneous coronary intervention; UFH, unfractionated heparin.

**Supplementary Table 5. Cochrane Collaboration’s tool for assessing the risk of bias for the included trials.**

| **Trial** | **Year of publication** | **RANDOM SEQUENCE GENERATION** | **ALLOCATION CONCEALMENT** | **BLINDING OF PARTICIPANTS** | **BLINDING OF**  **OUTCOME ASSESSMENT** | **INCOMPLETE**  **OUTCOME DATA** | **SELECTIVE**  **REPORTING** | **OTHER SOURCES**  **OF BIAS** |
| --- | --- | --- | --- | --- | --- | --- | --- | --- |
| **BARI**^1^ | 2006 |  |  |  |  |  |  |  |
| **Blazek et al. (2013)**^2^ | 2013 |  |  |  |  |  |  |  |
| **Boudriot et al.**^3^ | 2011 |  |  |  |  |  |  |  |
| **CARDia**^4^ | 2010 |  |  |  |  |  |  |  |
| **EXCEL**^5^ | 2019 |  |  |  |  |  |  |  |
| **FAME 3**^6^ | 2022 |  |  |  |  |  |  |  |
| **FREEDOM**^7,8^ | 2012 |  |  |  |  |  |  |  |
| **Hong et al.**^9^ | 2004 |  |  |  |  |  |  |  |
| **NOBLE**^10^ | 2020 |  |  |  |  |  |  |  |
| **SYNTAX**^11,12^ | 2013 |  |  |  |  |  |  |  |
| **Blazek et al. (2015)**^13^ | 2015 |  |  |  |  |  |  |  |
| **VA CARDS**^14^ | 2013 |  |  |  |  |  |  |  |
|  | | | | + | Low Risk | | | |
|  |  |  |  | ? | Uncertain | | | |
|  |  |  |  | - | High Risk | | | |

**Supplementary Table 6. Subgroup analyses of the correlations between periprocedural myocardial infarction (pMI) and all-cause and cardiac mortality.**

| Analysis | Studies | Patients | Regression formula | Slope (95%CI) | R^2^ (95%CI) |
| --- | --- | --- | --- | --- | --- |
| pMI definition: cardiac biomarkers rise >5 times URL |  |  |  |  |  |
| - pMI and all-cause mortality | 5 | 6587 | -0.48+2.07 x RR, P=0.009 | 2.07 (1.00 – 3.14) | 0.93 (0.20 – 0.96) |
| - pMI and cardiac mortality | 5 | 6587 | 0.64+0.70 x RR, P=0.02 | 0.70 (0.20 – 1.19) | 0.87 (0.03 – 0.93) |
| pMI definition: cardiac biomarkers rise ≤5 times URL |  |  |  |  |  |
| - pMI and all-cause mortality | 4 | 1044 | 0.96-0.19x RR, P=0.75 | -0.19 (-2.44 – 2.06) | 0.06 (0.002 – 0.55) |
| - pMI and cardiac mortality | 2 | 341 | NA | NA | NA |
| pMI definition: CK-MB rise >5 times URL |  |  |  |  |  |
| - pMI and all-cause mortality | 4 | 5087 | -0.45+2.08 x RR, P=0.03 | 2.08 (0.56 – 3.61) | 0.95 (0.001 – 0.97) |
| - pMI and cardiac mortality | 4 | 5087 | 0.65+0.70 x RR, P=0.07 | 0.70 (-0.11 – 1.51) | 0.87 (0.001 – 0.94) |
| pMI definition: CK-MB rise ≤5 times URL |  |  |  |  |  |
| - pMI and all-cause mortality | 4 | 1044 | 0.96-0.19x RR, P=0.75 | -0.19 (-2.44 – 2.06) | 0.06 (0.003 – 0.55) |
| - pMI and cardiac mortality | 2 | 341 | NA | NA | NA |
| Multivessel coronary disease |  |  |  |  |  |
| - pMI and all-cause mortality | 5 | 5929 | -0.76+2.16 x RR, P=0.02 | 2.16 (0.69 – 3.64) | 0.88 (0.05 – 0.94) |
| - pMI and cardiac mortality | 4 | 5427 | 0.82+0.59 x RR, P=0.02 | 0.59 (0.19 – 1.00) | 0.95 (0.01 – 0.98) |
| Left main disease |  |  |  |  |  |
| - pMI and all-cause mortality | 4 | 5090 | 2.26-1.47 x RR, P=0.34 | -1.47 (-6.56 – 3.62) | 0.43 (0.002 – 0.74) |
| - pMI and cardiac mortality | 3 | 4889 | 0.39+1.02 x RR, P=0.59 | 1.02 (-16.35 – 18.39) | 0.36 (0.004 – 0.72) |
| Drug-eluting stents |  |  |  |  |  |
| - pMI and all-cause mortality | 10 | 9508 | -0.62+1.95 x RR, P=0.001 | 1.95 (1.01 – 2.90) | 0.74 (0.22 – 0.86) |
| - pMI and cardiac mortality | 7 | 8615 | 1.21+0.34 x RR, P=0.27 | 0.34 (-0.36 – 1.05) | 0.24 (0.001 – 0.61) |
| Study year: after year 2000 |  |  |  |  |  |
| - pMI and all-cause mortality | 10 | 9508 | -0.62+1.95 x RR, P=0.001 | 1.95 (1.01 – 2.90) | 0.74 (0.22 – 0.86) |
| - pMI and cardiac mortality | 7 | 8615 | 1.21+0.34 x RR, P=0.27 | 0.34 (-0.36 – 1.05) | 0.24 (0.001 – 0.61) |
| Follow up >1 year |  |  |  |  |  |
| - pMI & All-cause mortality | 8 | 9157 | -0.10+1.86 x RR, P<0.001 | 1.86 (1.31 – 2.40) | 0.92 (0.58 – 0.96) |
| - pMI & Cardiac mortality | 8 | 9157 | 1.07+0.43 x RR, P=0.149 | 0.43 (-0.20 – 1.06) | 0.31 (0.002 – 0.64) |
| Follow up ≤1 year |  |  |  |  |  |
| - pMI & All-cause mortality | 4 | 2392 | -2.83+3.37 x RR, P=0.09 | 3.37 (-1.41 – 8.15) | 0.82 (0.004 – 0.91) |
| - pMI & Cardiac mortality | 1 | 1500 | NA | NA | NA |
| Follow up >4 years |  |  |  |  |  |
| - pMI & All-cause mortality | 7 | 8959 | 0.79+0.46 x RR, P=0.13 | 0.46 (-0.19 – 1.12) | 0.40 (0.003 – 0.70) |
| - pMI & Cardiac mortality | 7 | 8959 | 1.67+-0.52 x RR, P=0.49 | -0.52 (-2.28 – 1.25) | 0.10 (0.002 – 0.51) |
| Follow up ≤4 years |  |  |  |  |  |
| - pMI & All-cause mortality | 5 | 2590 | -2.58+3.13 x RR, P<0.001 | 3.13 (2.60 – 3.67) | 0.99 (0.82 – 1.00) |
| - pMI & Cardiac mortality | 2 | 1698 | NA | NA | NA |
| PCI |  |  |  |  |  |
| - pMI and All-cause mortality | 12 | 5813 | 0.01+0.60 x rate, P=0.04 | 0.60 (0.07 – 1.14) | 0.38 (0.004 – 0.65) |
| - pMI and cardiac mortality | 9 | 5340 | 0.00+0.36 x rate, P=0.04 | 0.36 (0.06 – 0.66) | 0.54 (0.01 – 0.75) |
| CABG |  |  |  |  |  |
| - pMI and All-cause mortality | 12 | 5736 | 0.02-0.25 x rate, P=0.45 | -0.25 (-0.45 – 0.90) | 0.06 (0.001 – 0.39) |
| - pMI and cardiac mortality | 9 | 5317 | 0.01+0.07 x rate, P=0.46 | 0.07 (-0.14 – 0.27) | 0.08 (0.001 – 0.46) |

pMI, periprocedural myocardial infarction; URL, upper reference limit.

**Supplementary Table 7. Sensitivity analyses of the impact of study-level factors on the correlation between periprocedural myocardial infarction (pMI) and all-cause and cardiac mortality.**

| Correlated outcomes | Assumed correlation | Study-level factor | pMI  RR (95%CI), P-value | Mortality  IRR (95%CI), P-value |
| --- | --- | --- | --- | --- |
| pMI & all-cause mortality | 0.9 | Age | -0.19 (-2.98 – 2.59), P=0.89 | -0.14 (-0.45 – 0.16), P=0.35 |
|  | 0.5 | Age | -0.11 (-2.93 – 2.71), P=0.94 | -0.15 (-0.44 – 0.14), P=0.30 |
|  | 0.1 | Age | -0.09 (-2.93 – 2.75), P=0.95 | -0.15 (-0.44 – 0.13), P=0.28 |
| pMI & cardiac mortality | 0.9 | Age | -0.13 (-2.89 – 2.63), P=0.93 | 0.00 (-0.20 – 0.20), P=0.99 |
|  | 0.5 | Age | -0.04 (-2.85 – 2.77), P=0.98 | -0.01 (-0.19 – 0.17), P=0.89 |
|  | 0.1 | Age | -0.10 (-2.94 – 2.75), P=0.95 | -0.02 (-0.19 – 0.16), P=0.86 |
| pMI & all-cause mortality | 0.9 | Sex | 0.11 (-0.42 – 0.65), P=0.68 | -0.09 (-0.13 – 0.03), P=0.08 |
|  | 0.5 | Sex | 0.07 (-0.50 – 0.63), P=0.81 | -0.09 (-0.13 – 0.02), P=0.22 |
|  | 0.1 | Sex | -0.02 (-0.59 – 0.55), P=0.94 | -0.09 (-0.13 – 0.01), P=0.34 |
| pMI & cardiac mortality | 0.9 | Sex | -0.06 (-0.63 – 0.51), P=0.84 | -0.02 (-0.06 – 0.02), P=0.32 |
|  | 0.5 | Sex | -0.04 (-0.60 – 0.53), P=0.90 | -0.02 (-0.06 – 0.02), P=0.25 |
|  | 0.1 | Sex | -0.04 (-0.61 – 0.53), P=0.88 | -0.02 (-0.06 – 0.02), P=0.24 |
| pMI & all-cause mortality | 0.9 | Diabetes | -0.02 (-0.18 – 0.15), P=0.84 | 0.01 (-0.01 – 0.03), P=0.09 |
|  | 0.5 | Diabetes | -0.01 (-0.17 – 0.16), P=0.93 | 0.01 (-0.01 – 0.02), P=0.07 |
|  | 0.1 | Diabetes | 0.01 (-0.16 – 0.17), P=0.94 | 0.01 (-0.01 – 0.02), P=0.06 |
| pMI & cardiac mortality | 0.9 | Diabetes | -0.02 (-0.16 – 0.12), P=0.75 | 0.01 (-0.02 – 0.01), P=0.37 |
|  | 0.5 | Diabetes | -0.02 (-0.17 – 0.14), P=0.85 | 0.00 (-0.01 – 0.01), P=0.42 |
|  | 0.1 | Diabetes | 0.01 (-0.16 – 0.17), P=0.94 | 0.00 (-0.01 – 0.01), P=0.42 |
| pMI & all-cause mortality | 0.9 | LVEF | 0.03 (-1.02 – 1.07), P=0.96 | 0.00 (-0.06 – 0.06), P=0.95 |
|  | 0.5 | LVEF | 0.06 (-1.42 – 1.55), P=0.93 | 0.01 (-0.05 – 0.07), P=0.81 |
|  | 0.1 | LVEF | 0.00 (-1.58 – 1.59), P=1.00 | 0.01 (-0.05 – 0.07), P=0.78 |
| pMI & cardiac mortality | 0.9 | LVEF | -0.37 (-1.78 – 1.04), P=0.60 | 0.02 (-0.06 – 0.10), P=0.66 |
|  | 0.5 | LVEF | -0.08 (-1.64 – 1.47), P=0.92 | 0.02 (-0.04 – 0.07), P=0.58 |
|  | 0.1 | LVEF | -0.02 (-1.61 – 1.57), P=0.98 | 0.02 (-0.04 – 0.07), P=0.57 |

IRR, incidence rate ratio; pMI, periprocedural myocardial infarction; RR, relative risk.

**Supplementary Figure 1. Preferred Reporting Items for Systematic Reviews and Meta-Analyses (PRISMA) flow diagram.**

**Identification of studies via databases and registers**

Records removed *before screening*:

Duplicate records removed

(n = 1744)

Records identified from:

Databases (n =7177)

-Ovid MEDLINE: 2078

-Ovid EMBASE: 4658

-Cochrane (Wiley): 441

Registers (n = 0)

**Identification**

Records screened

(n = 5433)

Records excluded

(n = 5389)

Reports sought for retrieval

(n = 44)

Reports not retrieved

(n = 0)

**Screening**

Reports excluded:

Non-randomized trial (n = 7)

Comparing balloon angioplasty versus CABG (n = 5)

Later follow-up available (n = 5)

Not original study (n = 2)

Not reporting data on periprocedural MI (n = 13)

Reports assessed for eligibility

(n = 44)

Studies included in review

(n = 12)

Reports of included studies

(n = 12)

**Included**

**Supplementary Figure 2. Subgroup analysis of the trials that required a raise in cardiac biomarkers (CK-MB or Troponin) >5 times the upper reference limits to diagnose periprocedural myocardial infarction (pMI) for the association between the relative risk (RR) for the surrogate end point of periprocedural myocardial infarction and the incidence rate ratio (IRR) for the true end points of all-cause or cardiac mortality**. The green area represents the 95%CI for the regression line (red), and circle sizes are proportionate to the number of observations. IRR, incident rate ratio; MI, myocardial infarction, RR relative risk.

**
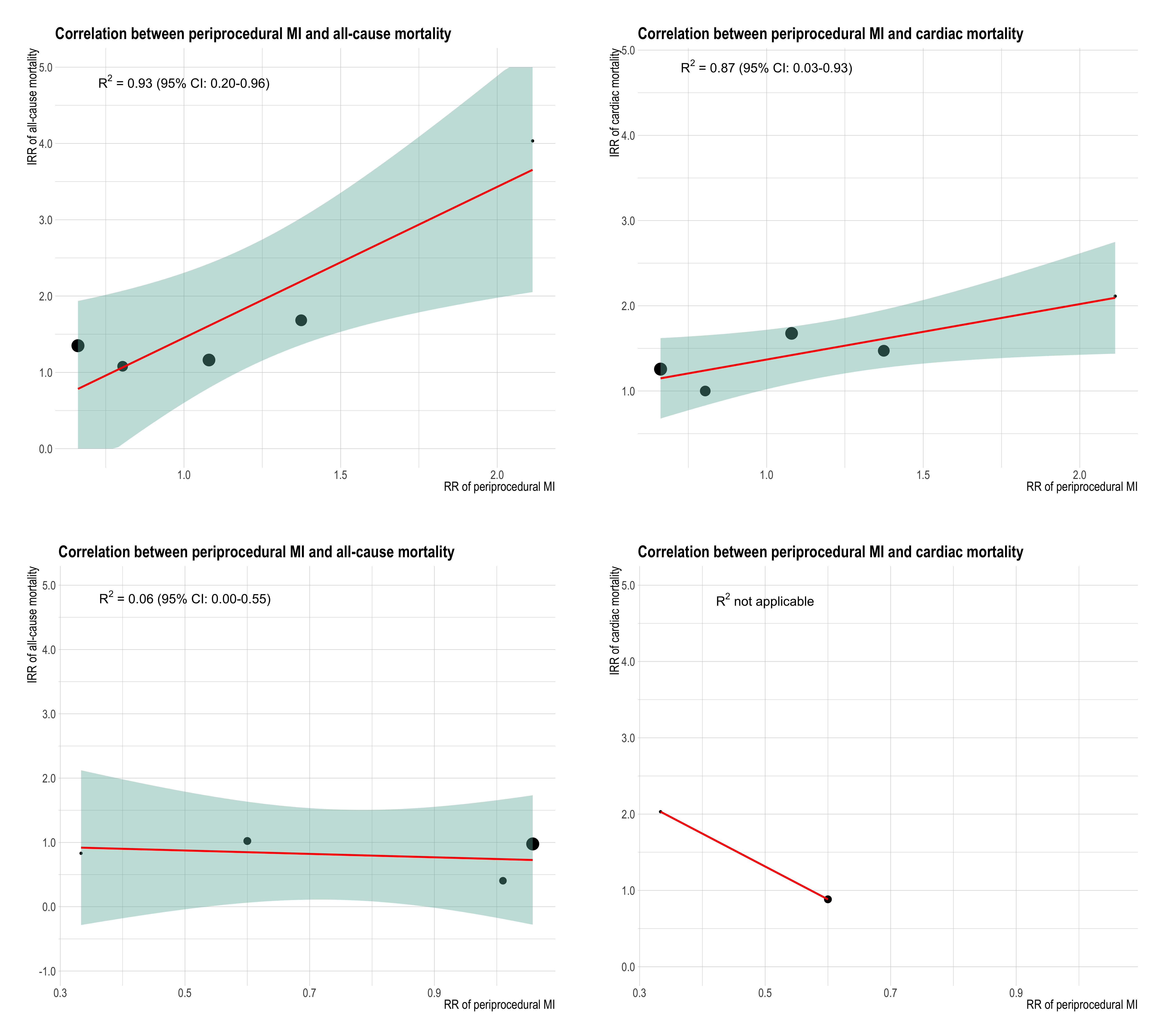
**

**Supplementary Figure 3. Subgroup analysis of the trials that required a raise in cardiac biomarkers (CK-MB or Troponin) ≤5 times the upper reference limits to diagnose periprocedural myocardial infarction (pMI) for the association between the relative risk (RR) for the surrogate end point of periprocedural myocardial infarction and the incidence rate ratio (IRR) for the true end points of all-cause or cardiac mortality**. The green area represents the 95%CI for the regression line (red), and circle sizes are proportionate to the number of observations. IRR, incident rate ratio; MI, myocardial infarction, RR relative risk.

**
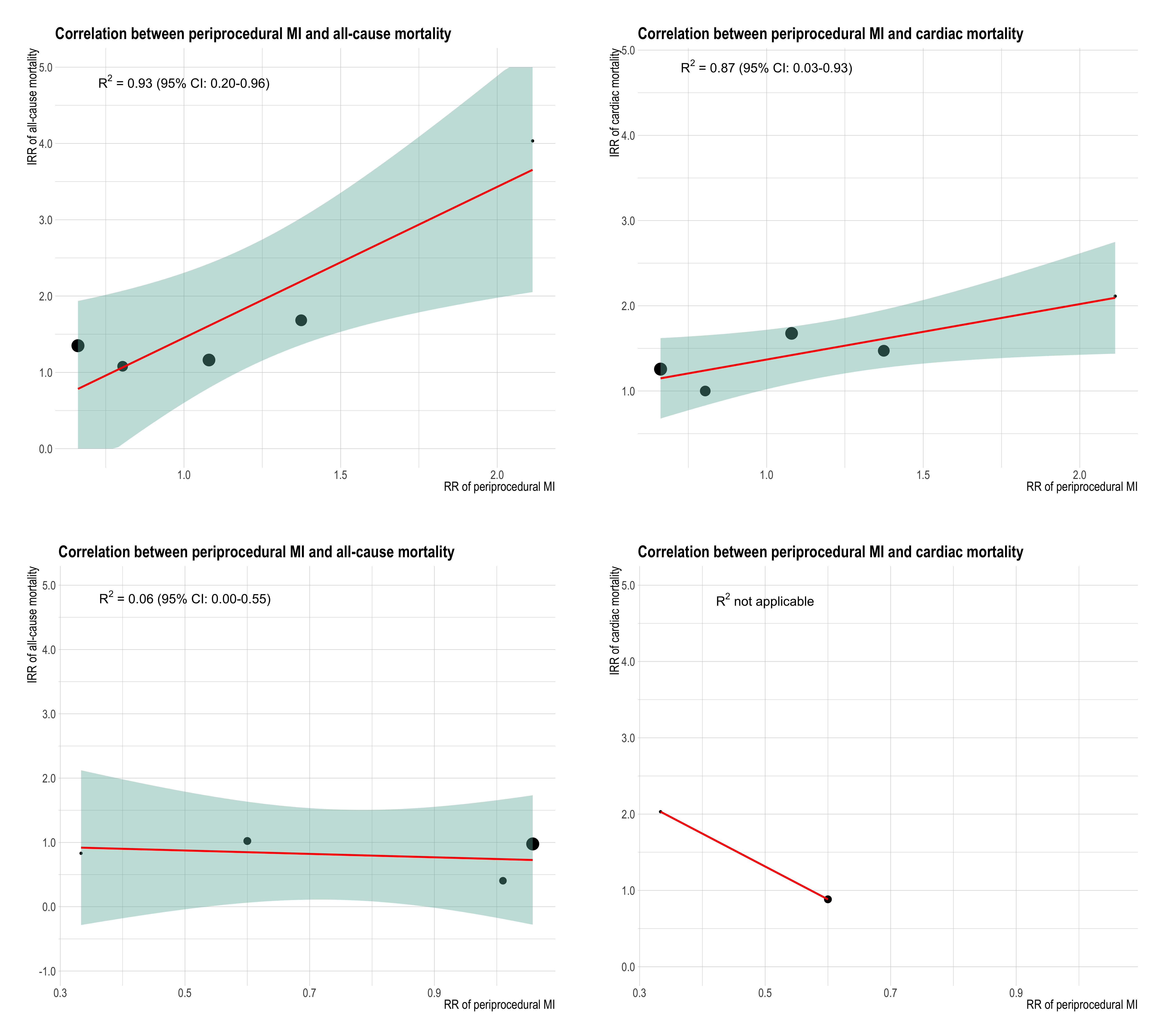
**

**Supplementary Figure 4. Subgroup analysis of the trials that required a rise in CK-MB >5 times the upper reference limits to diagnose periprocedural myocardial infarction (pMI) for the association between the relative risk (RR) for the surrogate endpoint of pMI and the incidence rate ratio (IRR) of the true endpoint for all-cause or cardiac mortality.** The green area represents the 95%CI for the regression line (red), and circle sizes are proportionate to the number of observations. IRR, incident rate ratio; pMI, periprocedural myocardial infarction; MI, myocardial infarction; RR relative risk.

**
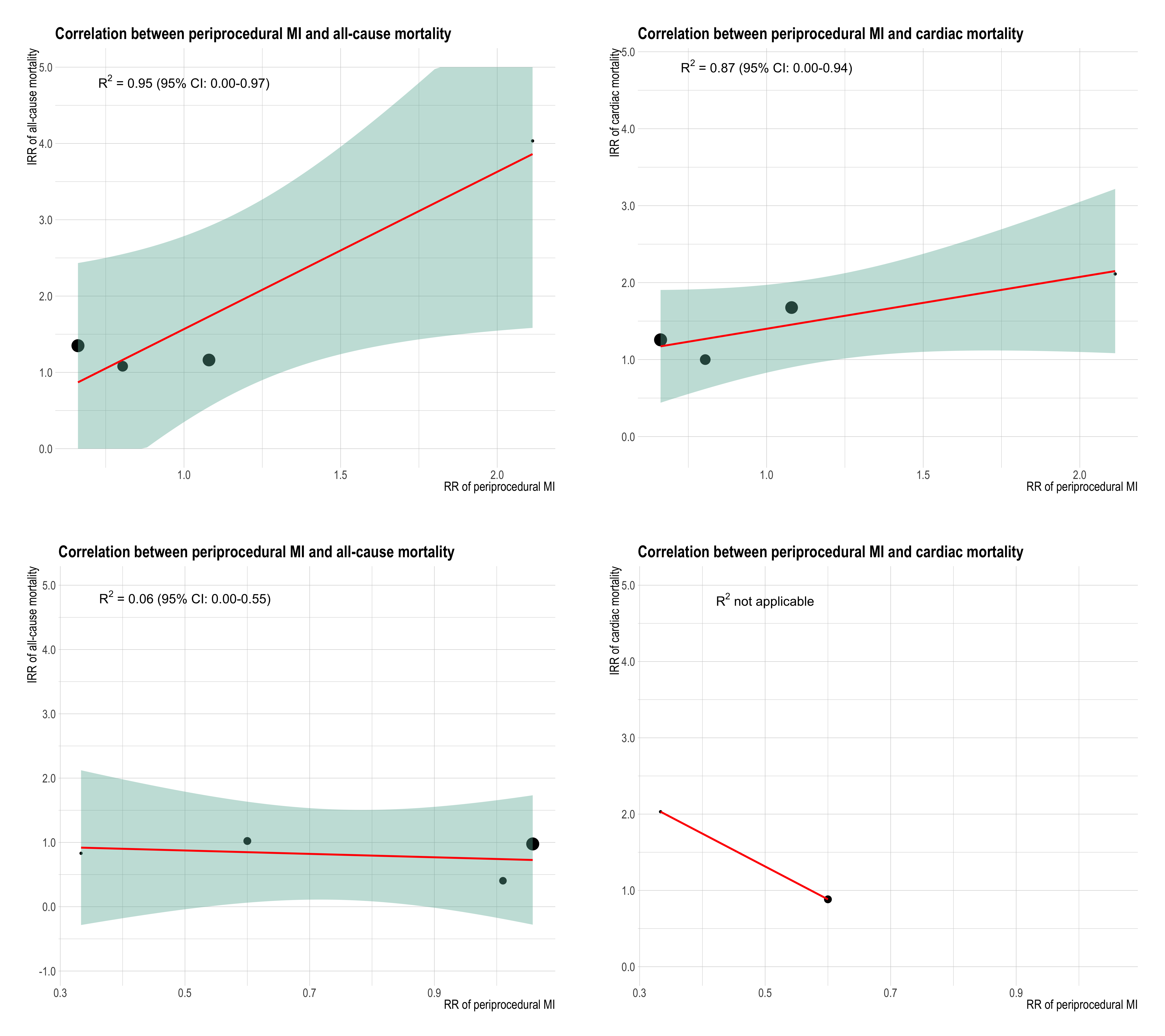
**

**Supplementary Figure 5. Subgroup analysis of the trials that required a rise in CK-MB ≤5 times the upper reference limits to diagnose periprocedural myocardial infarction (pMI) for the association between the relative risk (RR) for the surrogate endpoint of pMI and the incidence rate ratio (IRR) of the true endpoint for all-cause or cardiac mortality.** The green area represents the 95%CI for the regression line (red), and circle sizes are proportionate to the number of observations. IRR, incident rate ratio; pMI, periprocedural myocardial infarction; MI, myocardial infarction; RR relative risk.

**
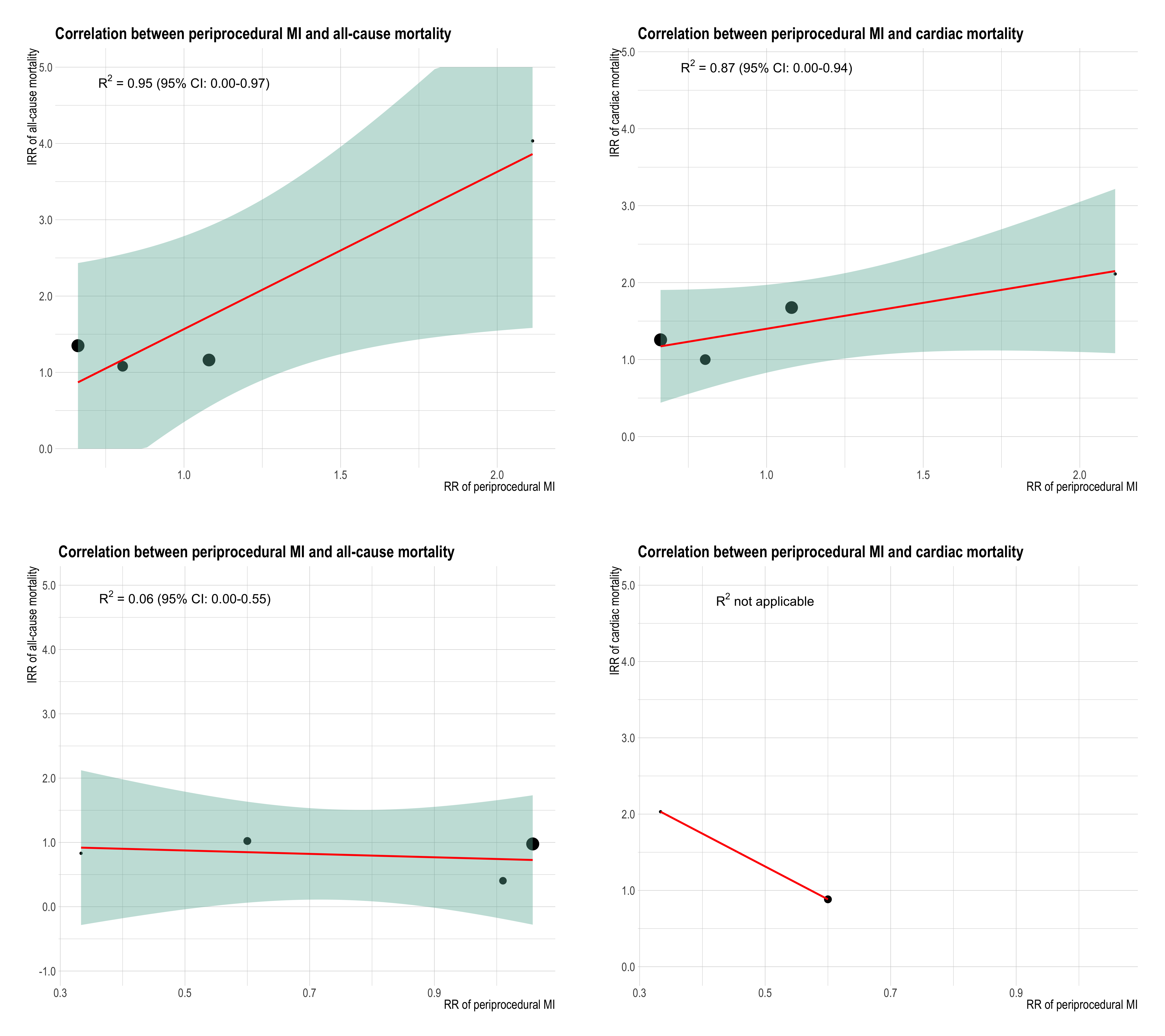
**

**Supplementary Figure 6. Subgroup analysis of the trials that included only patients with multivessel disease for the association between the relative risk (RR) for the surrogate endpoint of periprocedural myocardial infarction (pMI) and the incidence rate ratio (IRR) of the true endpoint for all-cause or cardiac mortality.** The green area represents the 95%CI for the regression line (red), and circle sizes are proportionate to the number of observations. IRR, incident rate ratio; pMI, periprocedural myocardial infarction; MI, myocardial infarction; RR, relative risk.

**
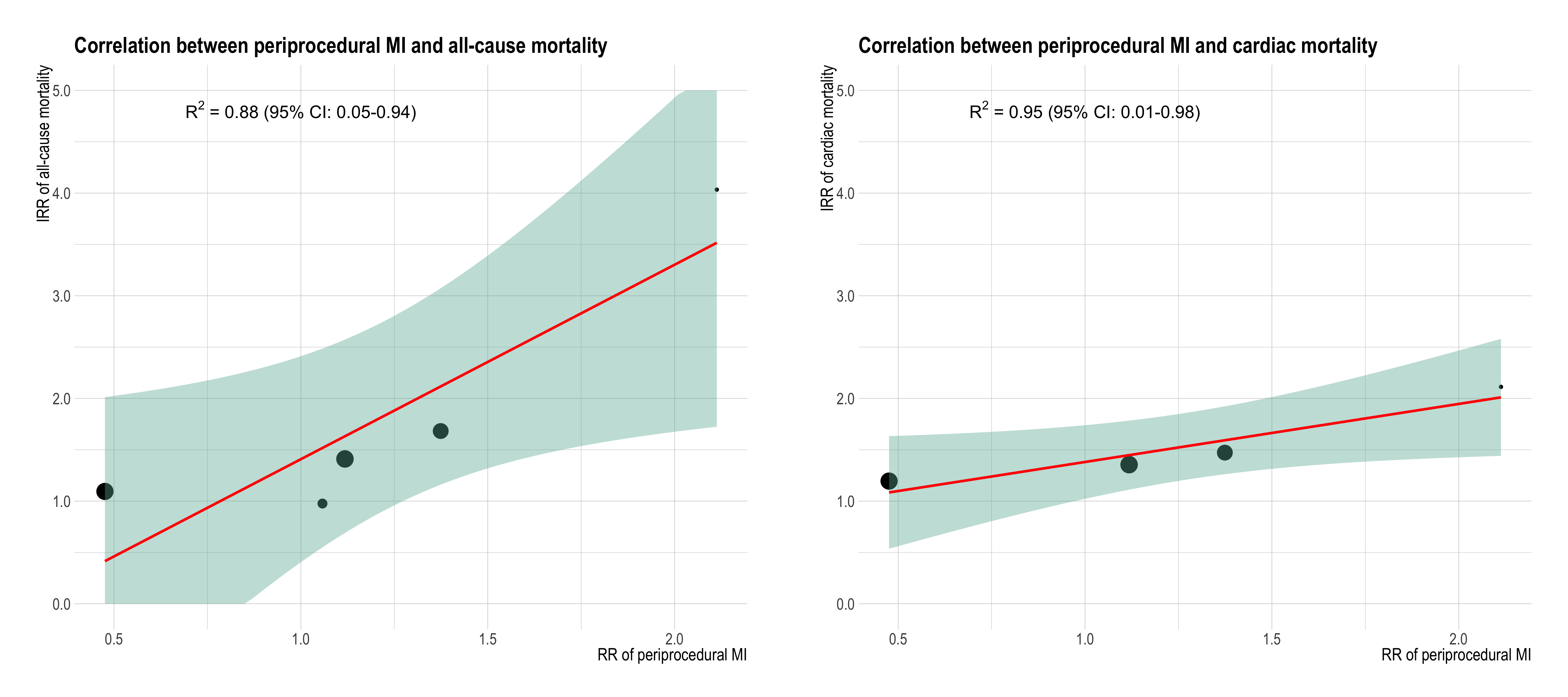
**

**Supplementary Figure 7. Subgroup analysis of the trials that included only patients with left main disease for the association between the relative risk (RR) for the surrogate endpoint of periprocedural myocardial infarction (pMI) and the incidence rate ratio (IRR) of the true endpoint for all-cause or cardiac mortality.** The green area represents the 95%CI for the regression line (red), and circle sizes are proportionate to the number of observations. IRR, incident rate ratio; pMI, periprocedural myocardial infarction; MI, myocardial infarction; RR, relative risk.

**
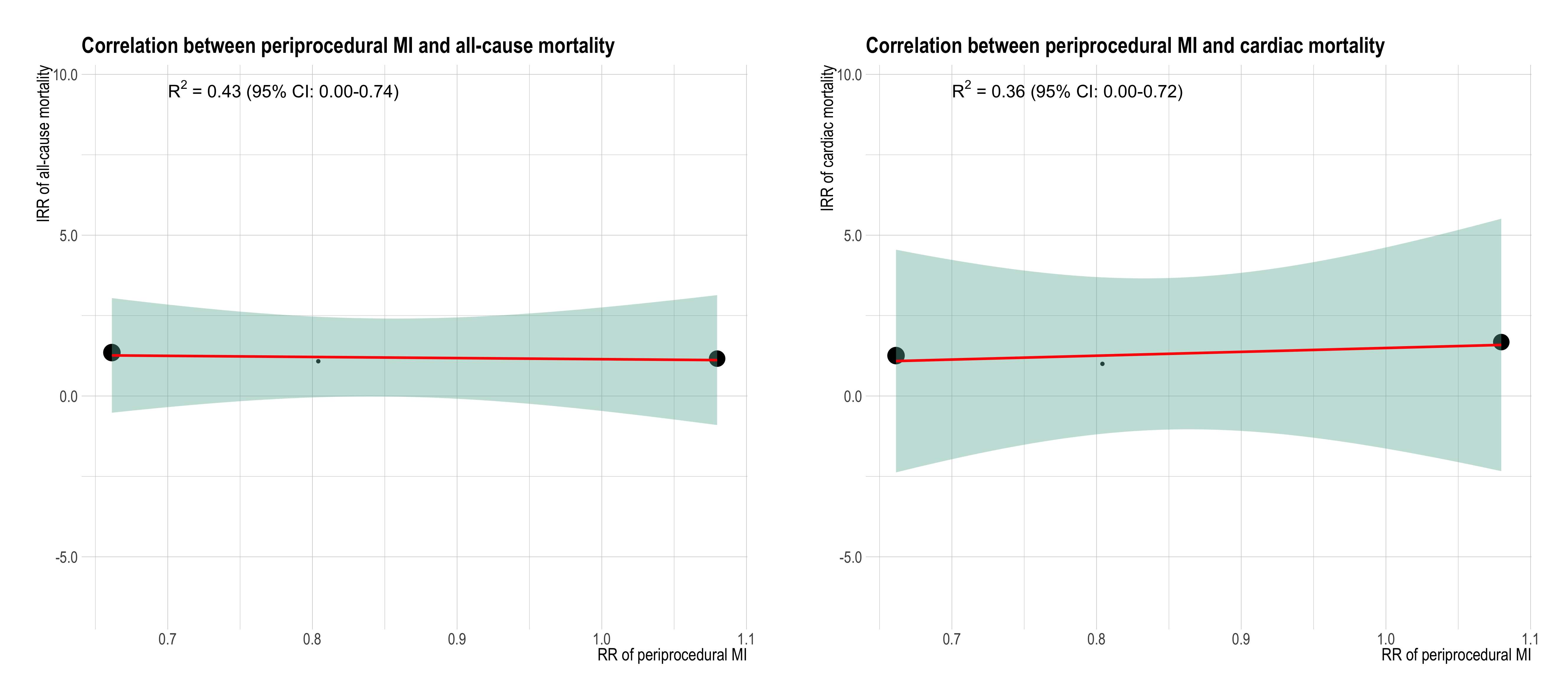
**

**Supplementary Figure 8. Subgroup analysis of the trials where only drug eluting stents (DES) were used for the association between the relative risk (RR) for the surrogate endpoint of periprocedural myocardial infarction (pMI) and the incidence rate ratio (IRR) of the true endpoint for all-cause or cardiac mortality.** The green area represents the 95%CI for the regression line (red), and circle sizes are proportionate to the number of observations. IRR, incident rate ratio; pMI, periprocedural myocardial infarction; MI, myocardial infarction; RR, relative risk.

**
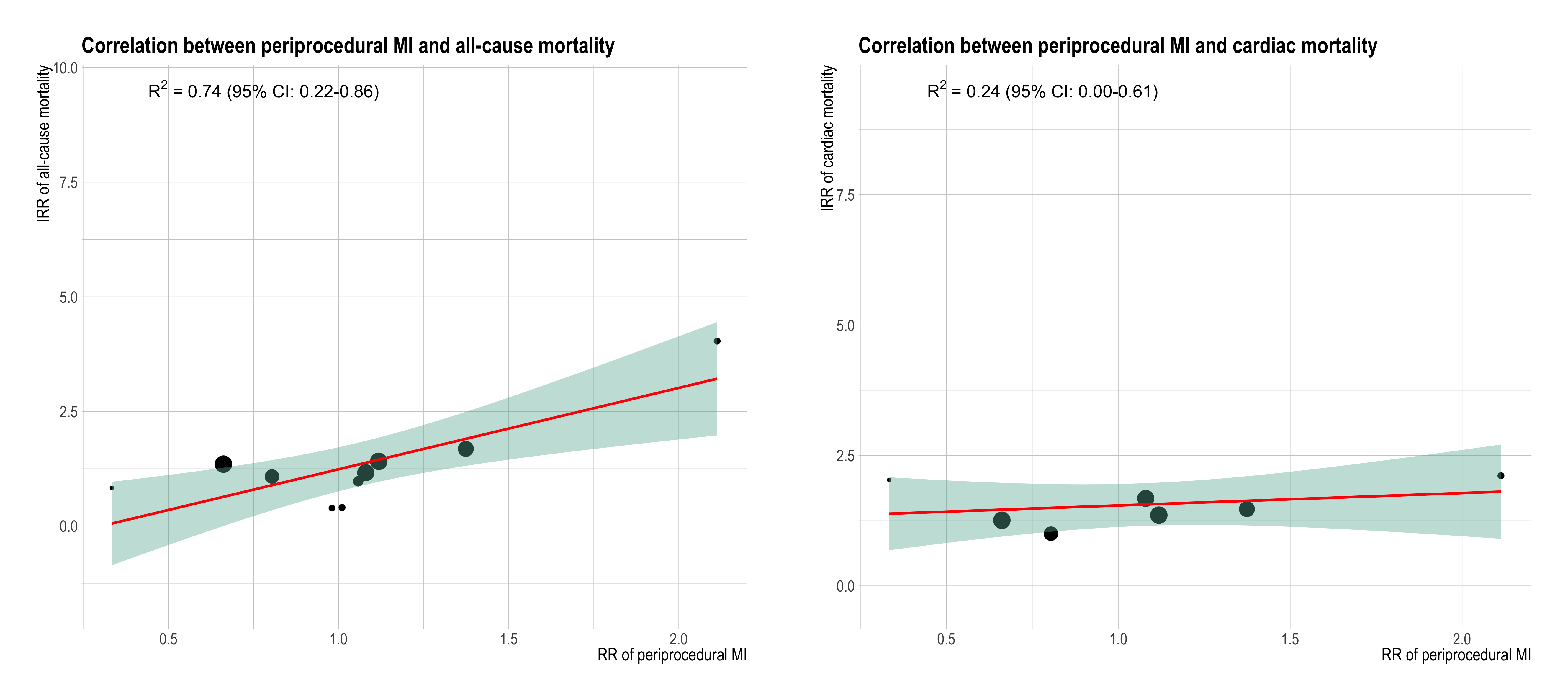
**

**Supplementary Figure 9. Subgroup analysis of the trials that were initiated after year 2000 for the association between the relative risk (RR) for the surrogate endpoint of periprocedural myocardial infarction (pMI) and the incidence rate ratio (IRR) of the true endpoint of all-cause or cardiac mortality.** The green area represents the 95%CI for the regression line (red), and circle sizes are proportionate to the number of observations. IRR, incident rate ratio; pMI, periprocedural myocardial infarction; MI, myocardial infarction; RR, relative risk.

**
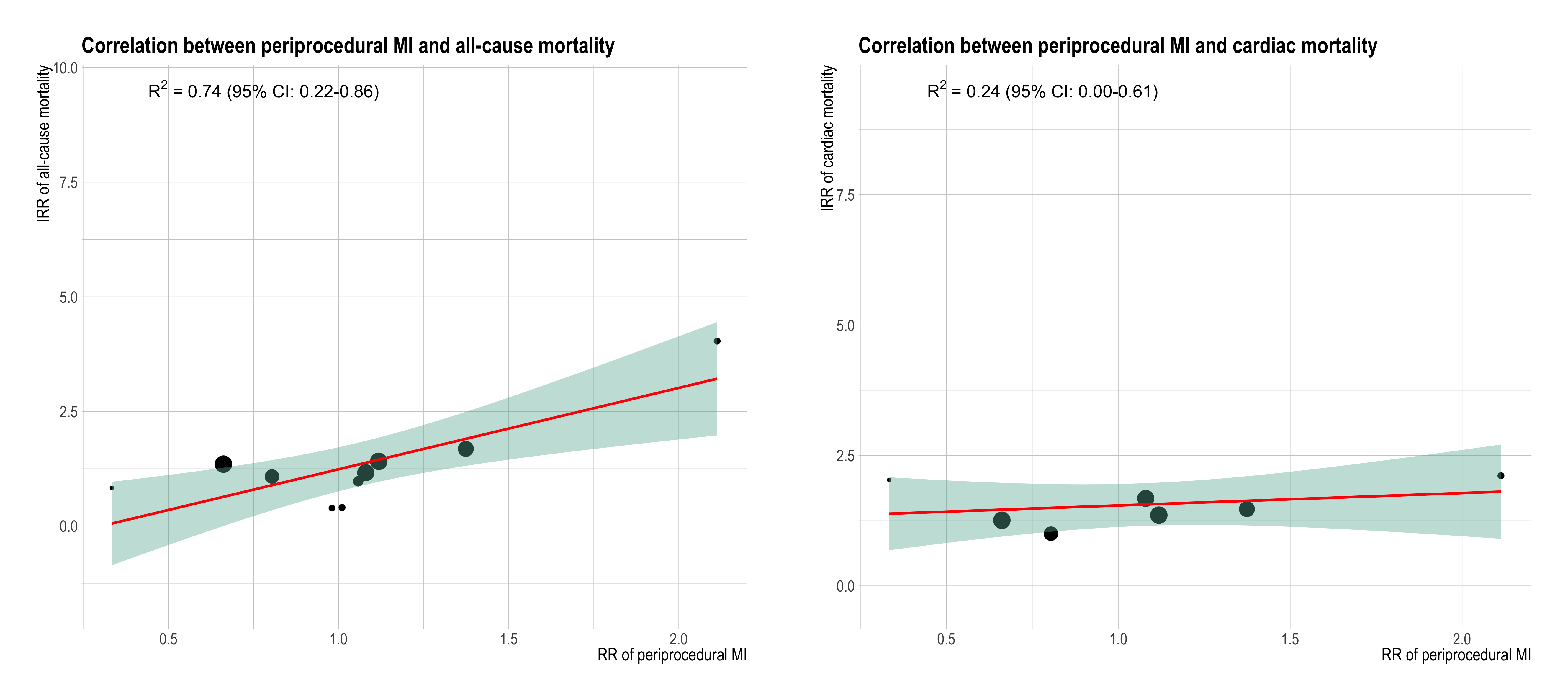
**

**Supplementary Figure 10. Subgroup analysis of the trials with follow-up duration >1 year for the association between the relative risk (RR) for the surrogate endpoint of periprocedural myocardial infarction (pMI) and the incidence rate ratio (IRR) for true endpoint of all-cause or cardiac mortality.** The green area represents the 95%CI for the regression line (red), and circle sizes are proportionate to the number of observations. IRR, incident rate ratio; pMI, periprocedural myocardial infarction; MI, myocardial infarction; RR, relative risk.

**
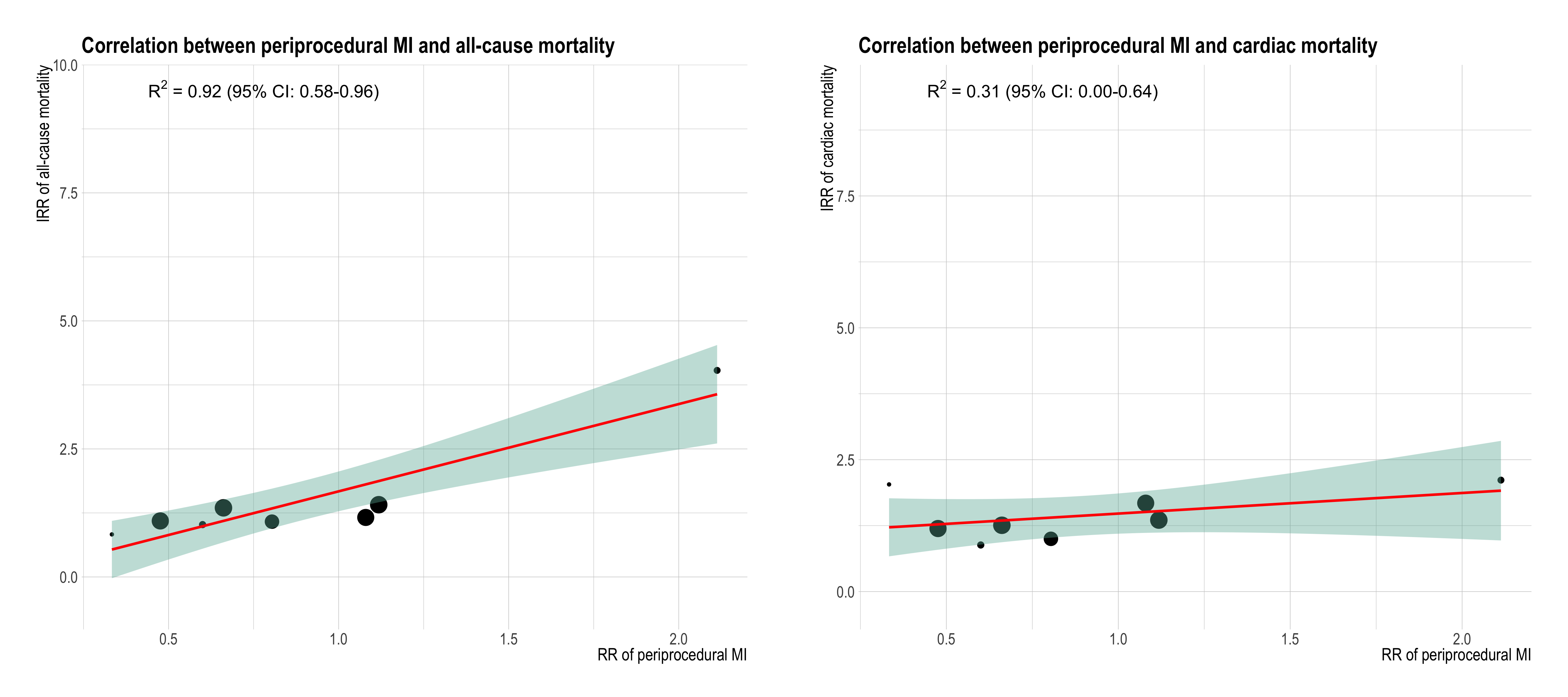
**

**Supplementary Figure 11. Subgroup analysis of the trials with follow-up duration ≤1 year for the association between the relative risk (RR) for the surrogate endpoint of periprocedural myocardial infarction (pMI) and the incidence rate ratio (IRR) for true endpoint of all-cause or cardiac mortality.** The green area represents the 95%CI for the regression line (red), and circle sizes are proportionate to the number of observations. IRR, incident rate ratio; pMI, periprocedural myocardial infarction; MI, myocardial infarction; RR, relative risk.


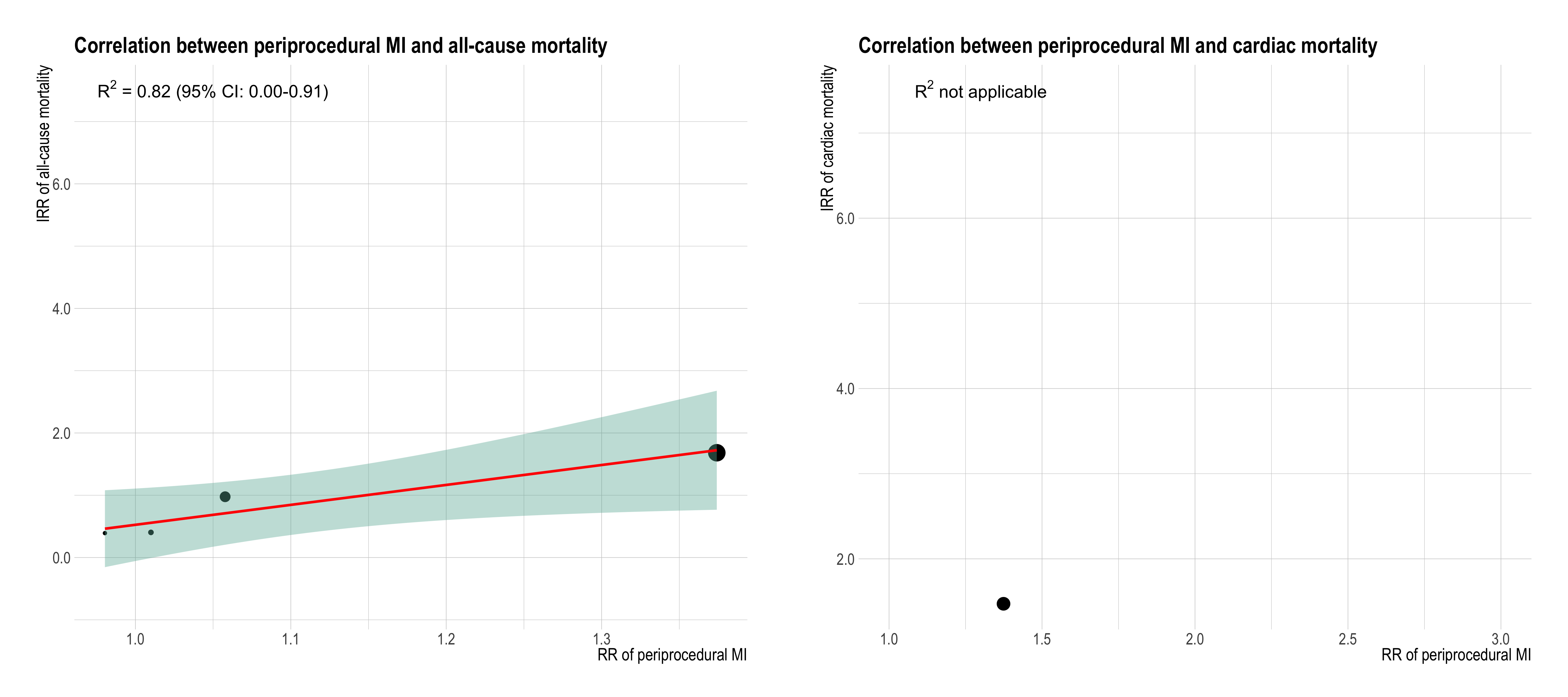


**Supplementary Figure 12. Subgroup analysis of the trials with follow-up duration >4 year for the association between the relative risk (RR) for the surrogate endpoint of periprocedural myocardial infarction (pMI) and the incidence rate ratio (IRR) for true endpoint of all-cause or cardiac mortality.** The green area represents the 95%CI for the regression line (red), and circle sizes are proportionate to the number of observations. IRR, incident rate ratio; pMI, periprocedural myocardial infarction; MI, myocardial infarction; RR, relative risk.

**
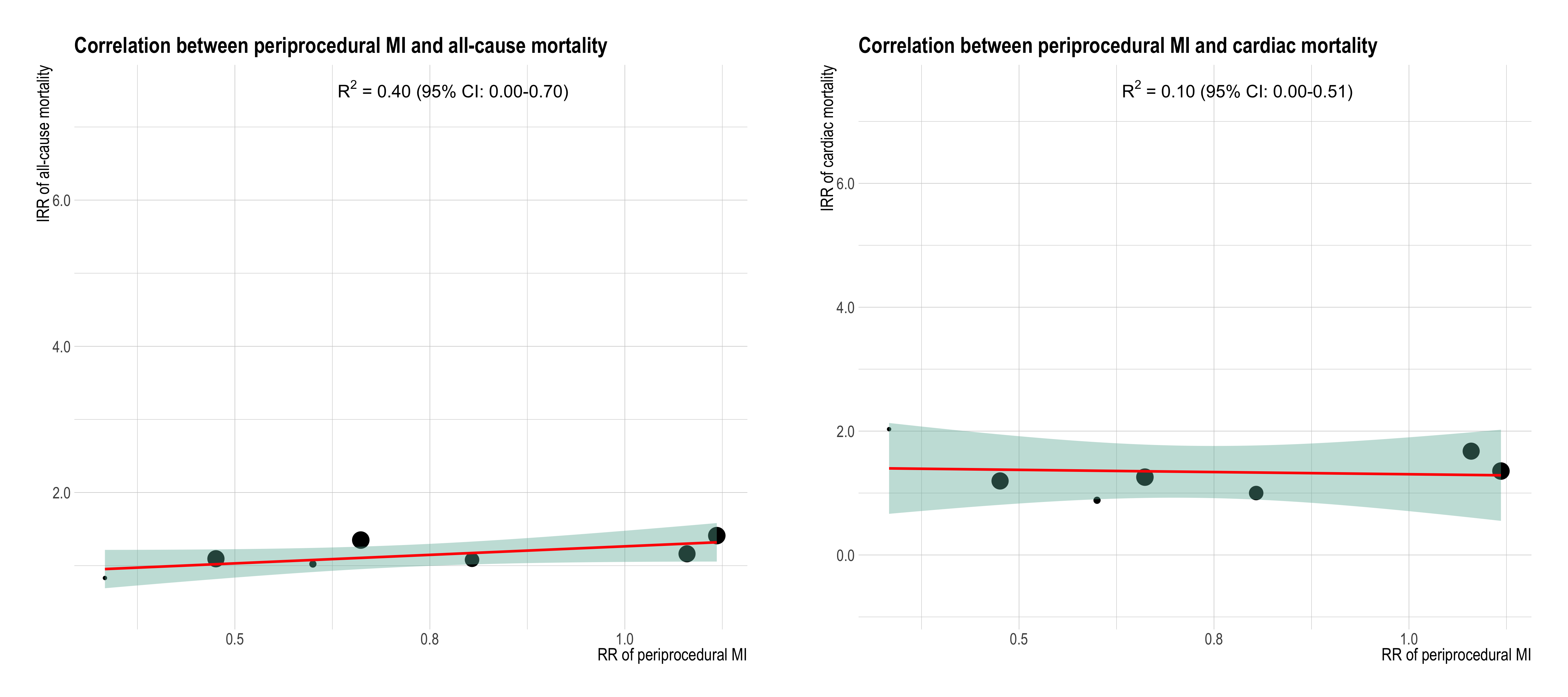
**

**Supplementary Figure 13. Subgroup analysis of the trials with follow-up duration ≤4 year for the association between the relative risk (RR) for the surrogate endpoint of periprocedural myocardial infarction (pMI) and the incidence rate ratio (IRR) for true endpoint of all-cause or cardiac mortality.** The green area represents the 95%CI for the regression line (red), and circle sizes are proportionate to the number of observations. IRR, incident rate ratio; pMI, periprocedural myocardial infarction; MI, myocardial infarction; RR, relative risk.


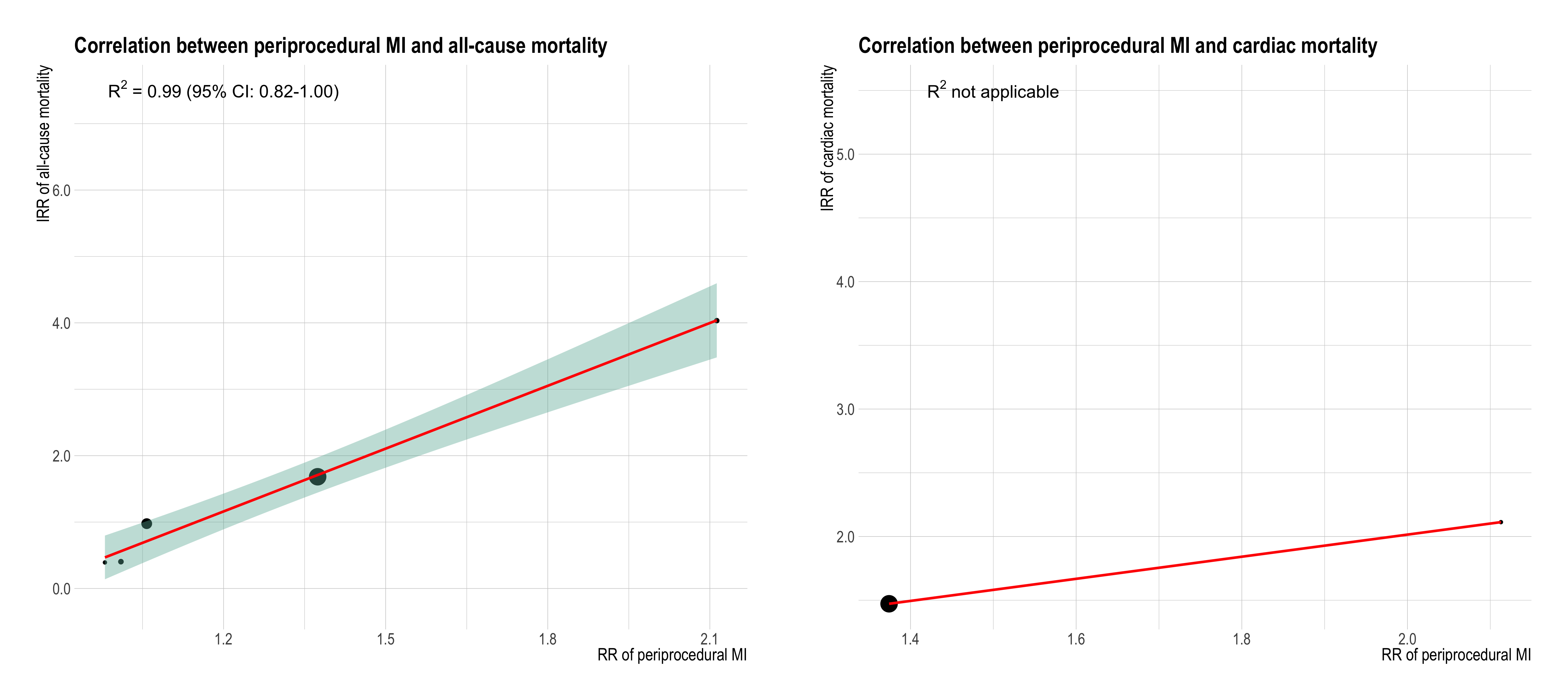


**Supplementary Figure 14. Correlation between the logarithm of the rate of the surrogate endpoint of periprocedural myocardial infarction (pMI) and the incidence rate (IR) for the true endpoint of all-cause mortality or cardiac mortality in PCI.** The green area represents the 95%CI for the regression line (red), and circle sizes are proportionate to the number of observations. IR, incident rate; pMI, periprocedural myocardial infarction; MI, myocardial infarction.


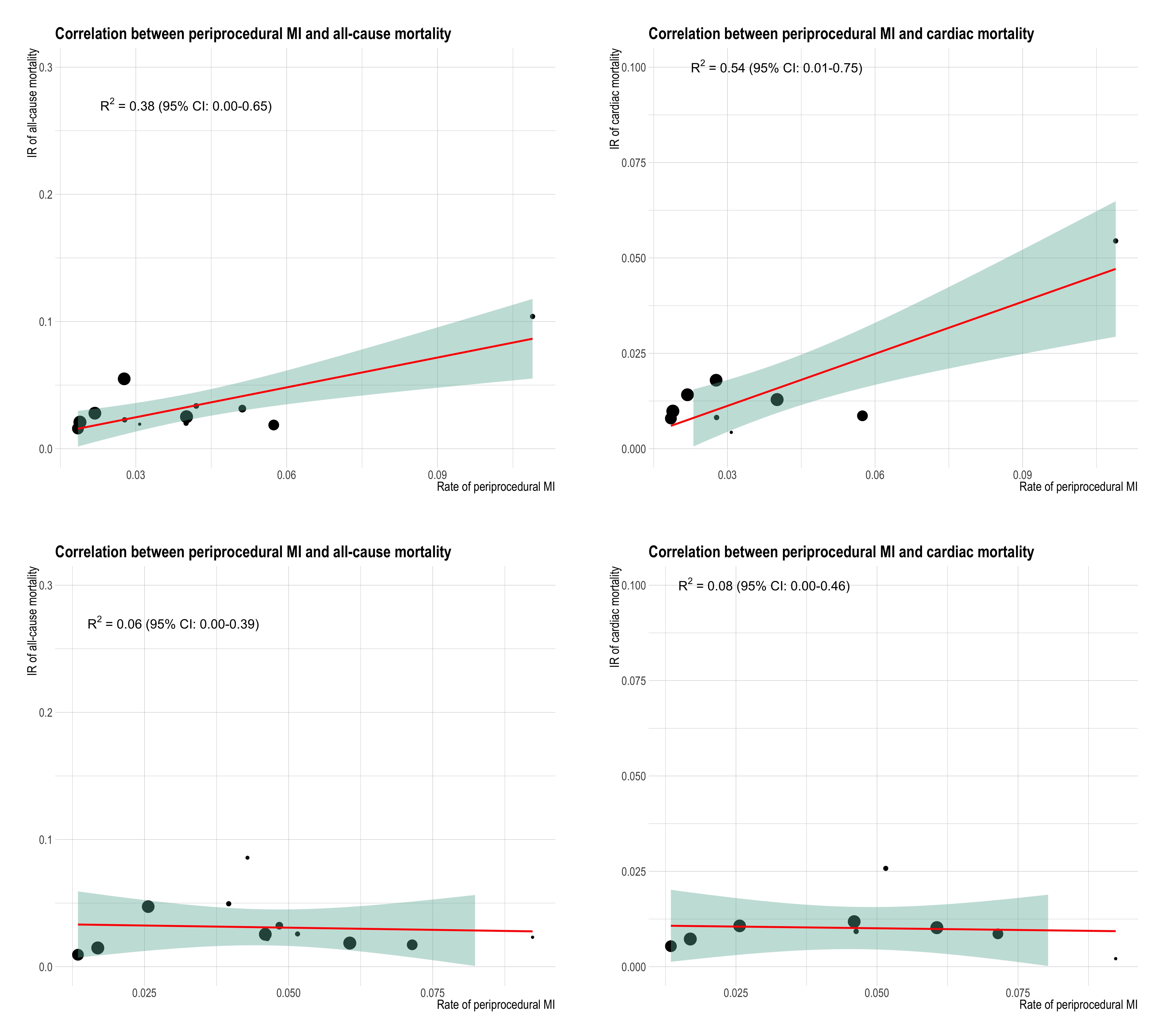


**Supplementary Figure 15. Correlation between the logarithm of the rate of the surrogate endpoint of periprocedural myocardial infarction (pMI) and the incidence rate (IR) for the true endpoint of all-cause mortality or cardiac mortality in CABG.** The green area represents the 95%CI for the regression line (red), and circle sizes are proportionate to the number of observations. IR, incident rate; pMI, periprocedural myocardial infarction; MI, myocardial infarction.


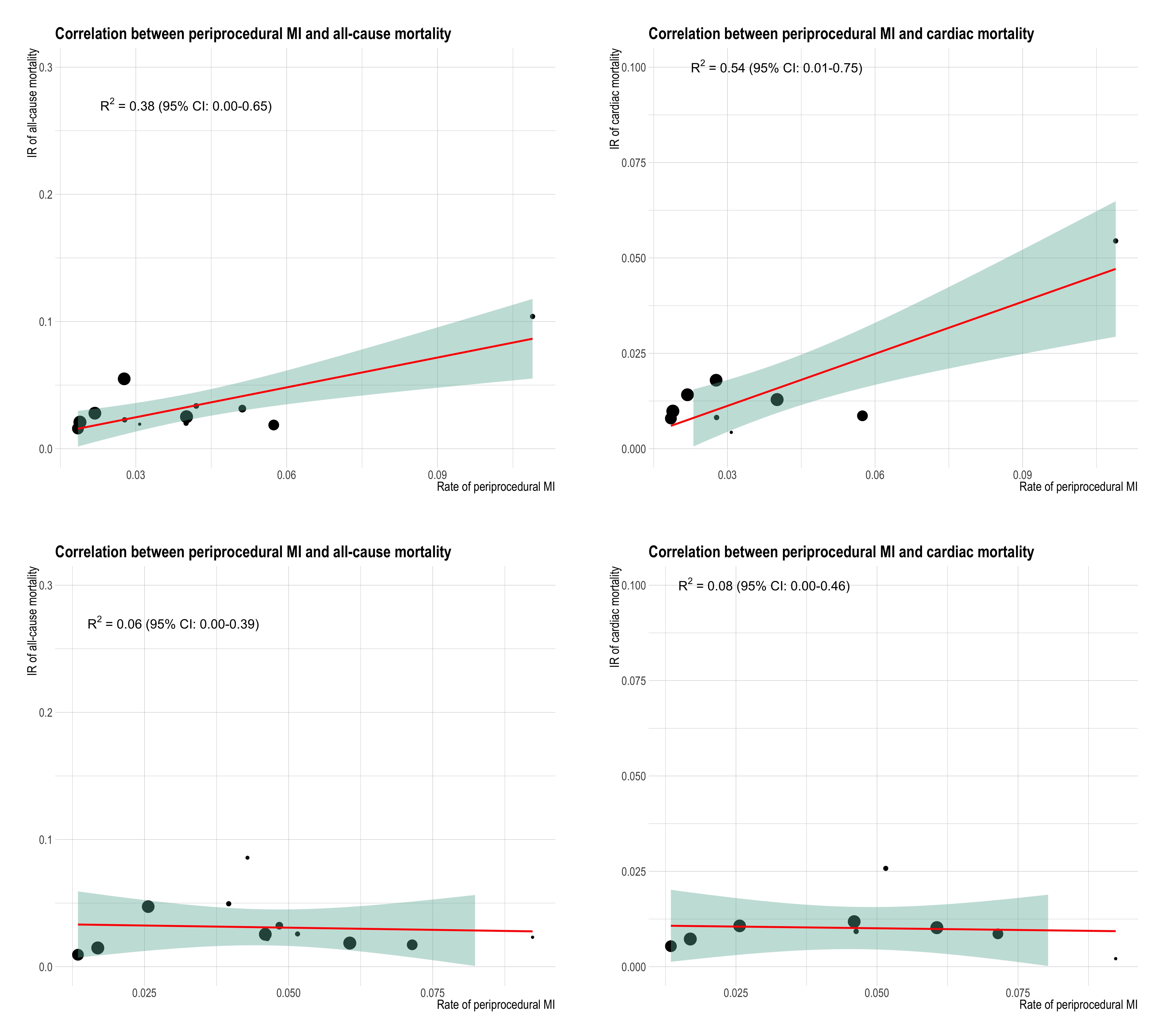


**Supplementary References**

1. The Final 10-Year Follow-Up Results From the BARI Randomized Trial. *J Am Coll Cardiol*. 2007;4915:1600-1606. doi:10.1016/j.jacc.2006.11.048

2. Blazek S, Holzhey D, Jungert C, Borger MA, Fuernau G, Desch S, et al. Comparison of Bare-Metal Stenting With Minimally Invasive Bypass Surgery for Stenosis of the Left Anterior Descending Coronary Artery. *JACC Cardiovasc Interv*. 2013;61:20-26. doi:10.1016/j.jcin.2012.09.008

3. Boudriot E, Thiele H, Walther T, Liebetrau C, Boeckstegers P, Pohl T, et al. Randomized Comparison of Percutaneous Coronary Intervention With Sirolimus-Eluting Stents Versus Coronary Artery Bypass Grafting in Unprotected Left Main Stem Stenosis. *J Am Coll Cardiol*. 2011;575:538-545. doi:10.1016/j.jacc.2010.09.038

4. Kapur A, Hall RJ, Malik IS, Qureshi AC, Butts J, de Belder M, et al. Randomized Comparison of Percutaneous Coronary Intervention With Coronary Artery Bypass Grafting in Diabetic Patients. *J Am Coll Cardiol*. 2010;555:432-440. doi:10.1016/j.jacc.2009.10.014

5. Stone GW, Kappetein AP, Sabik JF, Pocock SJ, Morice M-C, Puskas J, et al. Five-Year Outcomes after PCI or CABG for Left Main Coronary Disease. *N Engl J Med*. 2019;38119:1820-1830. doi:10.1056/NEJMoa1909406

6. Fearon WF, Zimmermann FM, De Bruyne B, Piroth Z, van Straten AHM, Szekely L, et al. Fractional Flow Reserve–Guided PCI as Compared with Coronary Bypass Surgery. *N Engl J Med*. 2022;3862:128-137. doi:10.1056/NEJMoa2112299

7. Farkouh ME, Domanski M, Sleeper LA, Siami FS, Dangas G, Mack M, et al. Strategies for Multivessel Revascularization in Patients with Diabetes. *N Engl J Med*. 2012;36725:2375-2384. doi:10.1056/NEJMoa1211585

8. Farkouh ME, Domanski M, Dangas GD, Godoy LC, Mack MJ, Siami FS, et al. Long-Term Survival Following Multivessel Revascularization in Patients With Diabetes. *J Am Coll Cardiol*. 2019;736:629-638. doi:10.1016/j.jacc.2018.11.001

9. Hong SJ, Lim D-S, Seo HS, Kim Y-H, Shim WJ, Park CG, et al. Percutaneous coronary intervention with drug-eluting stent implantation vs. minimally invasive direct coronary artery bypass (MIDCAB) in patients with left anterior descending coronary artery stenosis. *Catheter Cardiovasc Interv*. 2005;641:75-81. doi:10.1002/ccd.20238

10. Holm NR, Mäkikallio T, Lindsay MM, Spence MS, Erglis A, Menown IBA, et al. Percutaneous coronary angioplasty versus coronary artery bypass grafting in the treatment of unprotected left main stenosis: updated 5-year outcomes from the randomised, non-inferiority NOBLE trial. *The Lancet*. 2020;39510219:191-199. doi:10.1016/S0140-6736(19)32972-1

11. Mohr FW, Morice M-C, Kappetein AP, Feldman TE, Ståhle E, Colombo A, et al. Coronary artery bypass graft surgery versus percutaneous coronary intervention in patients with three-vessel disease and left main coronary disease: 5-year follow-up of the randomised, clinical SYNTAX trial. *The Lancet*. 2013;3819867:629-638. doi:10.1016/S0140-6736(13)60141-5

12. Thuijs DJFM, Kappetein AP, Serruys PW, Mohr F-W, Morice M-C, Mack MJ, et al. Percutaneous coronary intervention versus coronary artery bypass grafting in patients with three-vessel or left main coronary artery disease: 10-year follow-up of the multicentre randomised controlled SYNTAX trial. *The Lancet*. 2019;39410206:1325-1334. doi:10.1016/S0140-6736(19)31997-X

13. Blazek S, Rossbach C, Borger MA, Fuernau G, Desch S, Eitel I, et al. Comparison of Sirolimus-Eluting Stenting With Minimally Invasive Bypass Surgery for Stenosis of the Left Anterior Descending Coronary Artery. *JACC Cardiovasc Interv*. 2015;81:30-38. doi:10.1016/j.jcin.2014.08.006

14. Kamalesh M, Sharp TG, Tang XC, Shunk K, Ward HB, Walsh J, et al. Percutaneous Coronary Intervention Versus Coronary Bypass Surgery in United States Veterans With Diabetes. *J Am Coll Cardiol*. 2013;618:808-816. doi:10.1016/j.jacc.2012.11.044
